# Global, Regional, and National Burden of Myocarditis in 204 Countries and Territories From 1990 to 2019: Updated Systematic Analysis

**DOI:** 10.2196/46635

**Published:** 2024-01-11

**Authors:** Qingyu Kong, Xue Xu, Meng Li, Xiao Meng, Cuifen Zhao, Xiaorong Yang

**Affiliations:** 1 Department of Pediatrics Qilu Hospital of Shandong University Jinan, Shandong China; 2 Department of Cardiology Qilu Hospital of Shandong University Jinan, Shandong China; 3 Clinical Epidemiology Unit Qilu Hospital of Shandong University Jinan China; 4 Clinical Research Center of Shandong University Qilu Hospital Cheeloo College of Medicine Jinan China

**Keywords:** myocarditis, global burden, temporal trend, systematic analysis, incidence, mortality, disability-adjusted life years

## Abstract

**Background:**

Myocarditis is characterized by high disability and mortality, and imposes a severe burden on population health globally. However, the latest global magnitude and secular trend of myocarditis burden have not been reported.

**Objective:**

This study aimed to delineate the epidemiological characteristics of myocarditis burden globally for optimizing targeted prevention and research.

**Methods:**

Based on the Global Burden of Disease Study 2019, the myocarditis burden from 1990 to 2019 was modeled using the Cause of Death Ensemble tool, DisMod-MR, and spatiotemporal Gaussian regression. We depicted the epidemiology and trends of myocarditis by sex, age, year, region, and sociodemographic index (SDI). R program version 4.2.1 (R Project for Statistical Computing) was applied for all statistical analyses, and a 2-sided P-value of <.05 was considered statistically significant.

**Results:**

The number of incident cases (1,268,000) and deaths (32,450) associated with myocarditis in 2019 increased by over 1.6 times compared with the values in 1990 globally. On the other hand, the age-standardized incidence rate (ASIR) and age-standardized mortality rate (ASMR) decreased slightly from 1990 to 2019. The disability-adjusted life years (DALYs) decreased slightly in the past 3 decades, while the age-standardized DALY rate (ASDR) decreased greatly from 18.29 per 100,000 person-years in 1990 to 12.81 per 100,000 person-years in 2019. High SDI regions always showed a more significant ASIR. The ASIR slightly decreased in all SDI regions between 1990 and 2019. Middle SDI regions had the highest ASMR and ASDR in 2019. Low SDI regions had the lowest ASMR and ASDR in 2019. The age-standardized rates (ASRs) of myocarditis were higher among males than among females from 1990 to 2019 globally. All ASRs among both sexes had a downward trend, except for the ASMR among males, which showed a stable trend, and females had a more significant decrease in the ASDR than males. Senior citizens had high incident cases and deaths among both sexes in 2019. The peak numbers of DALYs for both sexes were noted in the under 1 age group in 2019. At the national level, the estimated annual percentage changes in the ASRs had significant negative correlations with the baseline ASRs in 1990.

**Conclusions:**

Globally, the number of incident cases and deaths associated with myocarditis have increased significantly. On the other hand, the ASRs of myocarditis showed decreasing trends from 1990 to 2019. Males consistently showed higher ASRs of myocarditis than females from 1990 to 2019 globally. Senior citizens gradually predominated in terms of myocarditis burden. Policymakers should establish targeted control strategies based on gender, region, age, and SDI; strengthen aging-related health research; and take notice of the changes in the epidemic characteristics of myocarditis.

## Introduction

Cardiovascular diseases can lead to high disability and mortality, which impose severe burdens on population health globally [1,2]. Among cardiovascular diseases, myocarditis has variable clinical presentations and outcomes, including chest pain with uncomplicated clinical manifestations, new onset or worsening heart failure, chronic dilated cardiomyopathy, and sudden death [3]. Myocarditis caused up to 9% of sudden deaths among cardiovascular events in athletes in the United States [4]. Moreover, 16% to 20% of cases of sudden infant death syndrome were documented to be associated with myocarditis [5-7]. Globally, 46,490 deaths were estimated to be due to myocarditis in 2017 [8]. Myocarditis characterized by high mortality represents an enormous public health burden.

Data for specific clinical settings are available to estimate the burden of myocarditis. Data showed that 3% of cases of chest pain in the emergency department were attributed to acute myocarditis and pericarditis [9]. In a prospective registry of northeastern Italy, autopsy studies showed a 12% incidence of myocarditis among young people who died suddenly [10]. The prevalence of myocarditis was reported to be 1.14% among patients with advanced cancers after therapy with immune checkpoint inhibitors (ICIs) [11]. The prevalence rate of myocarditis in hospitalized patients infected with COVID-19 was reported to be 2.4 per 1000 patients, considering definite or probable cases [12]. Hospital mortality was found to be much higher among patients with myocarditis and COVID-19 than among those with myocarditis but without COVID-19 [13]. The nationwide incidence of myocarditis in Israel due to the BNT162b2 mRNA COVID-19 vaccine was 2.7 per 100,000 persons [14]. In the last 2 decades, the diagnosis of myocarditis has become reasonably accurate among patients, including those from low-risk populations, through the use of noninvasive examinations, including high-sensitive troponin levels and cardiac magnetic resonance imaging (CMRI) [15,16]. In the United States, the incidence of myocarditis showed a gradual increase from 95 (in 2005) to 144 (in 2014) per 1 million persons [17]. It is quite clear that many factors can influence the global burden of myocarditis.

The Global Burden of Disease Study (GBD) 2019 is the most recent data set for evaluating the epidemiological levels and trends of 369 diseases along with 87 risk factors globally, and it has incorporated new data sets and modeling strategies compared with the GBD 2017 [18,19]. For example, the only country-level covariate of myocarditis used for the GBD 2019 was the Healthcare Access and Quality Index on excess mortality, which differs from the GBD 2017. Since the evaluation of the global burden of myocarditis using data from the GBD 2017, no comprehensive statistics on the global epidemiological trend of myocarditis have been calculated [8]. Due to the inclusion of more raw data and the application of more robust statistical methods in the GBD 2019, the results for myocarditis burden in the GBD 2019 differ dramatically from those in the GBD 2017, indicating that new statistics and analyses of the global burden of myocarditis need to be reported timely to guide its prevention and control. This research summarizes the latest epidemic characteristics of myocarditis, including incidence, death, disability-adjusted life years (DALYs), and changing trends by sex, age, and sociodemographic index (SDI), in 204 countries and territories from 1990 to 2019, using data from the GBD 2019. Our results will help to optimize the niche-targeting prevention and intervention of myocarditis that rely on concrete characteristics across the world.

## Methods

### Data Source

Previous studies have described the primary data explanations and analytic approaches of the GBD 2019 in detail [18-20]. Researchers can extract the reproducible statistical codes and analysis process online [21]. We briefly introduce the estimation methods specific to myocarditis. The Guidelines for Accurate and Transparent Health Estimates Reporting (GATHER) were followed to analyze the GBD database in every step (Report Checklist) [22]. Myocarditis was defined as a clinical diagnosis for the GBD 2019 estimation [2,19]. The International Classification of Diseases version 9 (ICD-9) and version 10 (ICD-10) codes were adopted to identify myocarditis. The determination of myocarditis was based on the disease codes 422-422.9 in ICD-9 and B33.2, I40-I41.9, and I51.4 in ICD-10. To assess the myocarditis burden, more than 250 primary data sources were screened out according to the GBD inclusion criteria. The myocarditis burden from 1990 to 2019 was modeled using the Cause of Death Ensemble tool, DisMod-MR, and spatiotemporal Gaussian regression. Data were obtained from the Institute for Health Metrics and Evaluation to characterize the global burden of myocarditis by sex and 5-year age group across the world in the past 3 decades. According to the SDI (a comprehensive index based on income per person, years of education, and fertility), the world is divided into 5 SDI regions (high, high-middle, middle, low-middle, and low) for assessing the myocarditis burden across different geographies. Furthermore, 204 countries and territories were divided into 21 GBD regions, including Western Europe, Tropical Latin America, and East Asia.

### Ethical Considerations

The GBD 2019 is a publicly available database, and all data were anonymous. Our study protocol was approved by the Institutional Review Board of Qilu Hospital of Shandong University with approval number KYLL-202011(KS)-239.

### Statistical Analysis

The burden of myocarditis was appraised based on the age-standardized incidence rate (ASIR), age-standardized mortality rate (ASMR), and age-standardized DALY rate (ASDR) by calendar year, sex, and region. The estimated annual percentage change (EAPC) was determined based on a regression calculation by fitting the natural logarithm of the age-standardized rate (ASR) with the historical year to characterize the long-term trend in the ASR of myocarditis burden [23]. The calculation formula is as follows:

ln (ASR) = α + β × historical year + ε **(1)**

The EAPC can be used to depict the changing trends of the ASR in a specific population and a certain time interval. The EAPC and its 95% CI are calculated using the following formula:

100 × (exp(β)-1) **(2)**

An increasing trend was recognized when the EAPC and the minimum of the 95% CI were positive. On the contrary, a decreasing trend was recognized when the EAPC and the maximum of the 95% CI were negative. Otherwise, the trend of the ASR was considered to be stable. The Spearman rank correlation with ρ coefficient was used to estimate the influence of the baseline ASR in 1990 and the SDI in 2019 all over the world on the EAPC in myocarditis burden. R program version 4.2.1 (R Project for Statistical Computing) was used for all statistical analyses, and a 2-sided *P*-value of <.05 was considered statistically significant.

## Results

### Global Burden and Temporal Trend of Myocarditis

**Figure 1:**
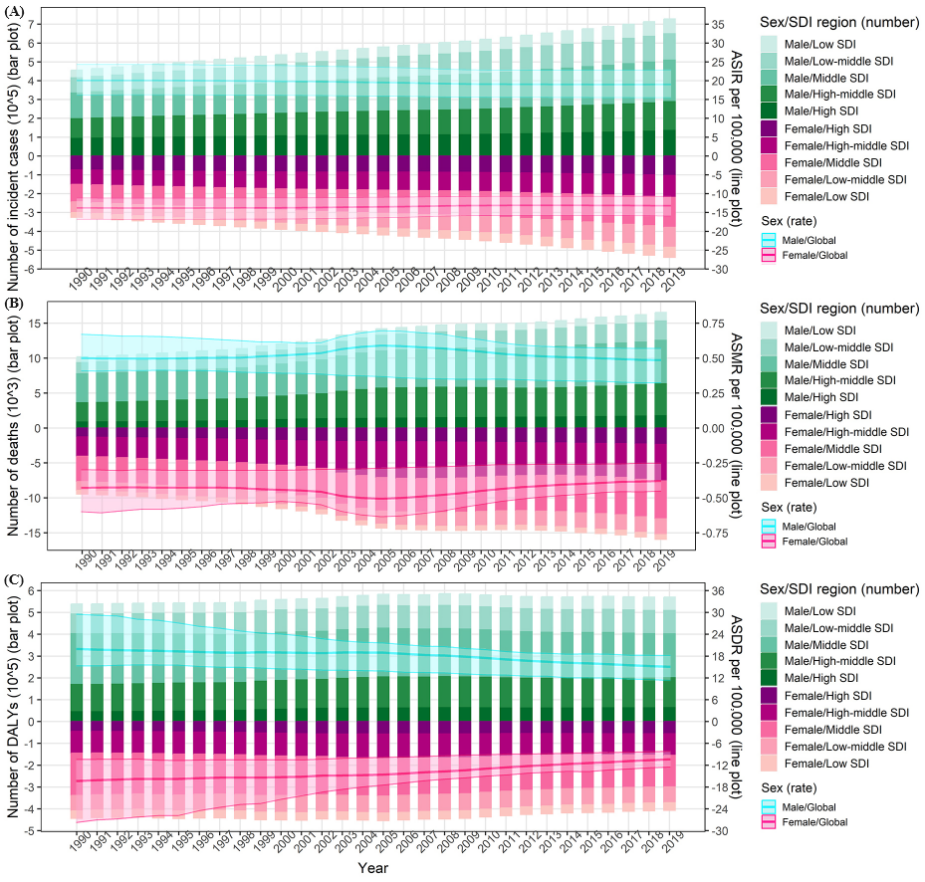
Absolute counts and age-standardized rates of the global myocarditis burden by sex/SDI region from 1990 to 2019. (A) Incidence; (B) Mortality; (C) DALYs. ASDR: age-standardized DALY rate; ASIR: age-standardized incidence rate; ASMR: age-standardized mortality rate; DALYs: disability-adjusted life years; SDI: sociodemographic index.

Across the world, the number of incident cases of myocarditis increased from 780,400 (95% uncertainty interval [UI] 620,600-951,200) in 1990 to 1,265,800 (95% UI 1,021,700-1,531,500) in 2019, with an increase of 62.20%. On the other hand, the ASIR decreased slightly from 16.74 (95% UI 13.46-20.34) to 16.00 (95% UI 13.01-19.28) per 100,000 person-years over the 3 decades, with an EAPC of −0.23 (95% CI −0.26 to −0.21) (Table 1; Figure 1). Simultaneously, the number of worldwide deaths caused by myocarditis increased from 19,620 (95% UI 15,690-26,770) in 1990 to 32,450 (95% UI 23,160-37,090) in 2019, with an increase of 65.39%. On the other hand, the ASMR decreased marginally from 0.46 (95% UI 0.38-0.60) to 0.43 (95% UI 0.31-0.50) per 100,000 person-years over the 3 decades, with an EAPC of −0.09 (95% CI −0.39 to 0.21) (Table 2; Figure 1). Globally, the DALYs decreased slightly. However, the ASDR decreased greatly from 18.29 (95% UI 13.81-27.58) per 100,000 person-years in 1990 to 12.81 (95% UI 10.53-14.72) per 100,000 person-years in 2019, with an EAPC of −1.19 (95% CI −1.33 to −1.04) (Table 3; Figure 1).

**Table 1:** Incidence and age-standardized incidence rate of myocarditis in 1990 and 2019, and the estimated annual percentage change from 1990 to 2019.

Characteristic		1990		2019		EAPC^a^ in the ASIR^b^ from 1990 to 2019, value (95% CI)
		ASIR/100,000 (95% UI^c^)	Incident cases×10^4^ (95% UI)	ASIR/100,000 (95% UI)	Incident cases×10^4^ (95% UI)	
**Global**		16.74 (13.46 to 20.34)	78.04 (62.06 to 95.12)	16.00 (13.01 to 19.28)	126.58 (102.17 to 153.15)	−0.23 (−0.26 to −0.21)
	Male	20.06 (16.21 to 24.31)	45.55 (36.39 to 55.72)	19.06 (15.51 to 22.89)	72.79 (58.52 to 87.92)	−0.25 (−0.27 to −0.22)
	Female	13.66 (11.04 to 16.67)	32.49 (25.87 to 39.72)	13.10 (10.62 to 15.84)	53.79 (43.56 to 65.14)	−0.23 (−0.27 to −0.20)
**SDI^d^ region**						
	High	18.72 (15.07 to 22.80)	17.26 (13.85 to 21.06)	17.75 (14.71 to 21.22)	24.54 (20.01 to 29.83)	−0.41 (−0.49 to −0.34)
	High-middle	16.58 (13.32 to 20.18)	18.29 (14.53 to 22.28)	16.01 (12.94 to 19.34)	26.96 (21.58 to 32.81)	−0.16 (−0.18 to −0.14)
	Middle	16.39 (13.25 to 19.96)	22.98 (18.20 to 28.36)	15.79 (12.79 to 19.10)	37.90 (30.26 to 46.21)	−0.16 (−0.17 to −0.14)
	Low-middle	15.93 (12.90 to 19.29)	13.71 (10.95 to 16.81)	15.68 (12.73 to 18.97)	24.52 (19.55 to 29.85)	−0.06 (−0.07 to −0.06)
	Low	15.35 (12.43 to 18.68)	5.77 (4.60 to 7.12)	15.29 (12.38 to 18.59)	12.60 (10.01 to 15.55)	−0.02 (−0.02 to −0.01)
**GBD^e^ region**						
	High-income Asia Pacific	20.66 (16.78 to 24.99)	3.76 (3.02 to 4.58)	20.07 (16.38 to 24.09)	5.48 (4.34 to 6.76)	−0.20 (−0.24 to −0.16)
	High-income North America	19.91 (16.01 to 24.29)	6.36 (5.08 to 7.78)	18.21 (15.37 to 21.45)	8.78 (7.33 to 10.35)	−0.83 (−1.01 to −0.65)
	Western Europe	17.56 (14.19 to 21.33)	8.14 (6.55 to 9.93)	17.47 (14.21 to 21.14)	10.95 (8.74 to 13.5)	−0.06 (−0.09 to −0.03)
	Australasia	16.51 (13.25 to 19.98)	0.36 (0.29 to 0.44)	16.45 (13.31 to 19.83)	0.62 (0.50 to 0.75)	−0.02 (−0.04 to −0.01)
	Southern Latin America	15.34 (12.43 to 18.64)	0.72 (0.58 to 0.88)	15.36 (12.45 to 18.66)	1.16 (0.94 to 1.41)	0.01 (0.00 to 0.02)
	Andean Latin America	14.05 (11.38 to 17.11)	0.40 (0.32 to 0.49)	14.07 (11.41 to 17.10)	0.84 (0.68 to 1.02)	0.01 (0.00 to 0.02)
	Tropical Latin America	15.76 (12.72 to 19.19)	1.90 (1.52 to 2.33)	15.72 (12.68 to 19.15)	3.68 (2.97 to 4.49)	−0.01 (−0.01 to −0.01)
	Central Latin America	15.06 (12.18 to 18.32)	1.84 (1.48 to 2.26)	14.97 (12.11 to 18.20)	3.64 (2.94 to 4.44)	−0.02 (−0.02 to −0.02)
	Caribbean	14.21 (11.50 to 17.19)	0.43 (0.35 to 0.52)	14.19 (11.48 to 17.16)	0.70 (0.57 to 0.86)	0.00 (0.00 to 0.00)
	Eastern Europe	17.64 (14.19 to 21.42)	4.27 (3.40 to 5.19)	17.74 (14.28 to 21.53)	4.61 (3.67 to 5.63)	0.02 (0.01 to 0.02)
	Central Europe	16.30 (13.13 to 19.83)	2.11 (1.68 to 2.58)	16.48 (13.27 to 20.05)	2.46 (1.96 to 3.02)	0.00 (−0.04 to 0.03)
	Central Asia	14.84 (11.86 to 17.95)	0.88 (0.70 to 1.07)	14.87 (11.88 to 17.98)	1.25 (0.99 to 1.53)	0.01 (0.01 to 0.01)
	North Africa and Middle East	12.01 (9.68 to 14.61)	3.14 (2.46 to 3.91)	12.05 (9.72 to 14.66)	6.41 (5.07 to 7.91)	0.02 (0.02 to 0.02)
	South Asia	16.28 (13.20 to 19.69)	13.40 (10.6 to 16.52)	16.18 (13.12 to 19.58)	26.00 (20.69 to 31.77)	−0.02 (−0.02 to −0.02)
	Southeast Asia	15.14 (12.27 to 18.41)	5.71 (4.54 to 7.03)	15.14 (12.26 to 18.38)	9.64 (7.65 to 11.76)	0.00 (−0.01 to 0.00)
	East Asia	17.92 (14.47 to 21.97)	19.2 (15.15 to 23.81)	16.86 (13.73 to 20.36)	28.35 (22.9 to 34.67)	−0.28 (−0.32 to −0.23)
	Oceania	15.30 (12.32 to 18.49)	0.08 (0.06 to 0.09)	15.30 (12.31 to 18.50)	0.16 (0.13 to 0.20)	0.00 (0.00 to 0.00)
	Western Sub-Saharan Africa	15.48 (12.54 to 18.87)	2.14 (1.71 to 2.63)	15.37 (12.45 to 18.71)	4.99 (3.96 to 6.15)	−0.03 (−0.03 to −0.02)
	Eastern Sub-Saharan Africa	15.26 (12.33 to 18.61)	2.00 (1.58 to 2.47)	15.25 (12.32 to 18.59)	4.43 (3.50 to 5.48)	−0.01 (−0.01 to −0.01)
	Central Sub-Saharan Africa	14.59 (11.72 to 17.82)	0.56 (0.44 to 0.69)	14.52 (11.67 to 17.70)	1.35 (1.06 to 1.67)	−0.02 (−0.02 to −0.01)
	Southern Sub-Saharan Africa	15.72 (12.72 to 19.06)	0.63 (0.51 to 0.78)	15.73 (12.72 to 19.08)	1.07 (0.85 to 1.31)	0.00 (0.00 to 0.01)

^a^EAPC: estimated annual percentage change.

^b^ASIR: age-standardized incidence rate.

^c^UI: uncertainty interval.

^d^SDI: sociodemographic index.

^e^GBD: Global Burden of Disease Study.

**Table 2:** Deaths and age-standardized mortality rate of myocarditis in 1990 and 2019, and the estimated annual percentage change from 1990 to 2019.

Characteristic		1990		2019		EAPC^a^ in the ASMR^b^ from 1990 to 2019, value (95% CI)
		ASMR/100,000 (95% UI^c^)	Deaths×10^3^ (95% UI)	ASMR/100,000 (95% UI)	Deaths×10^3^ (95% UI)	
**Global**		0.46 (0.38 to 0.60)	19.62 (15.69 to 26.77)	0.43 (0.31 to 0.50)	32.45 (23.16 to 37.09)	−0.09 (−0.39 to 0.21)
	Male	0.50 (0.41 to 0.67)	10.18 (8.02 to 14.38)	0.48 (0.32 to 0.57)	16.53 (11.21 to 19.24)	0.07 (−0.19 to 0.34)
	Female	0.42 (0.30 to 0.59)	9.44 (6.40 to 14.25)	0.38 (0.25 to 0.45)	15.92 (10.49 to 19.09)	−0.26 (−0.59 to 0.08)
**SDI^d^ region**						
	High	0.26 (0.21 to 0.33)	2.32 (1.87 to 2.93)	0.27 (0.21 to 0.31)	4.26 (3.13 to 4.92)	0.12 (−0.13 to 0.37)
	High-middle	0.59 (0.45 to 0.74)	5.50 (4.36 to 7.15)	0.56 (0.40 to 0.65)	9.82 (7.06 to 11.47)	−0.06 (−0.64 to 0.52)
	Middle	0.67 (0.54 to 0.94)	7.80 (6.16 to 11.9)	0.59 (0.35 to 0.72)	11.64 (7.38 to 13.95)	−0.07 (−0.31 to 0.16)
	Low-middle	0.39 (0.28 to 0.47)	2.85 (1.69 to 3.93)	0.41 (0.24 to 0.50)	5.06 (3.21 to 6.02)	0.48 (0.23 to 0.73)
	Low	0.31 (0.21 to 0.44)	1.14 (0.57 to 1.91)	0.24 (0.15 to 0.37)	1.66 (1.18 to 2.46)	−0.97 (−1.06 to −0.89)
**GBD^e^ region**						
	High-income Asia Pacific	0.33 (0.22 to 0.38)	0.53 (0.38 to 0.60)	0.21 (0.16 to 0.25)	0.78 (0.44 to 1.03)	−1.76 (−1.86 to −1.65)
	High-income North America	0.17 (0.14 to 0.25)	0.48 (0.40 to 0.73)	0.26 (0.18 to 0.30)	1.11 (0.73 to 1.30)	1.76 (1.18 to 2.35)
	Western Europe	0.25 (0.18 to 0.32)	1.20 (0.85 to 1.54)	0.34 (0.19 to 0.43)	3.33 (1.68 to 4.50)	1.63 (0.66 to 2.61)
	Australasia	0.36 (0.3 to 0.48)	0.07 (0.06 to 0.10)	0.30 (0.25 to 0.42)	0.12 (0.10 to 0.16)	−0.91 (−1.4 to −0.41)
	Southern Latin America	0.28 (0.19 to 0.35)	0.12 (0.09 to 0.15)	0.24 (0.19 to 0.32)	0.19 (0.15 to 0.25)	−0.64 (−0.76 to −0.51)
	Andean Latin America	0.27 (0.15 to 0.37)	0.06 (0.03 to 0.09)	0.12 (0.09 to 0.16)	0.07 (0.05 to 0.09)	−3.09 (−3.29 to −2.88)
	Tropical Latin America	0.28 (0.23 to 0.41)	0.31 (0.24 to 0.44)	0.30 (0.25 to 0.46)	0.67 (0.54 to 1.01)	0.14 (−0.23 to 0.51)
	Central Latin America	0.12 (0.09 to 0.13)	0.14 (0.11 to 0.17)	0.14 (0.09 to 0.17)	0.32 (0.22 to 0.40)	0.43 (0.30 to 0.57)
	Caribbean	0.38 (0.24 to 0.66)	0.12 (0.07 to 0.24)	0.36 (0.24 to 0.56)	0.17 (0.12 to 0.25)	−0.13 (−0.21 to −0.05)
	Eastern Europe	0.41 (0.24 to 0.53)	0.85 (0.55 to 1.06)	0.53 (0.33 to 0.67)	1.56 (0.89 to 2.05)	0.70 (0.56 to 0.83)
	Central Europe	1.05 (0.69 to 1.34)	1.24 (0.79 to 1.58)	1.12 (0.80 to 1.36)	2.25 (1.59 to 2.76)	−0.69 (−1.14 to −0.24)
	Central Asia	0.47 (0.38 to 0.64)	0.25 (0.21 to 0.35)	0.78 (0.61 to 1.11)	0.62 (0.48 to 0.95)	2.55 (1.75 to 3.35)
	North Africa and Middle East	0.52 (0.34 to 0.85)	1.60 (0.94 to 3.31)	0.36 (0.26 to 0.65)	1.72 (1.23 to 3.05)	−1.19 (−1.24 to −1.15)
	South Asia	0.24 (0.16 to 0.33)	1.38 (0.97 to 1.82)	0.21 (0.15 to 0.29)	2.83 (2.04 to 3.80)	−0.41 (−0.48 to −0.34)
	Southeast Asia	0.42 (0.28 to 0.53)	1.22 (0.84 to 1.88)	0.36 (0.23 to 0.44)	1.82 (1.26 to 2.36)	−0.56 (−0.75 to −0.36)
	East Asia	1.07 (0.81 to 1.68)	8.90 (7.10 to 13.44)	0.90 (0.53 to 1.11)	13.5 (7.91 to 16.74)	−0.16 (−0.50 to 0.19)
	Oceania	0.30 (0.17 to 0.44)	0.02 (0.01 to 0.03)	0.33 (0.18 to 0.50)	0.04 (0.02 to 0.07)	0.56 (0.47 to 0.64)
	Western Sub-Saharan Africa	0.41 (0.17 to 0.70)	0.34 (0.15 to 0.55)	0.24 (0.17 to 0.31)	0.55 (0.38 to 0.74)	−2.32 (−2.58 to −2.06)
	Eastern Sub-Saharan Africa	0.24 (0.11 to 0.37)	0.51 (0.20 to 0.96)	0.14 (0.05 to 0.33)	0.47 (0.19 to 0.90)	−1.89 (−2.02 to −1.75)
	Central Sub-Saharan Africa	0.35 (0.21 to 0.53)	0.15 (0.06 to 0.36)	0.24 (0.08 to 0.50)	0.19 (0.09 to 0.37)	−1.33 (−1.40 to −1.27)
	Southern Sub-Saharan Africa	0.30 (0.20 to 0.38)	0.12 (0.07 to 0.17)	0.22 (0.16 to 0.33)	0.14 (0.10 to 0.21)	−1.45 (−1.66 to −1.24)

^a^EAPC: estimated annual percentage change.

^b^ASMR: age-standardized mortality rate.

^c^UI: uncertainty interval.

^d^SDI: sociodemographic index.

^e^GBD: Global Burden of Disease Study.

**Table 3:** Disability-adjusted life years (DALYs) and age-standardized DALY rate of myocarditis in 1990 and 2019, and the estimated annual percentage change from 1990 to 2019.

Characteristic		1990		2019		EAPC^a^ in the ASDR^b^ from 1990 to 2019, value (95% CI)
		ASDR/100,000 (95% UI^c^)	DALYs^d^×10^4^ (95% UI)	ASDR/100,000 (95% UI)	DALYs×10^4^ (95% UI)	
**Global**		18.29 (13.81 to 27.58)	98.14 (70.70 to 157.9)	12.81 (10.53 to 14.72)	97.72 (80.38 to 112.68)	−1.19 (−1.33 to −1.04)
	Male	20.14 (15.47 to 29.74)	53.83 (39.73 to 84.54)	15.21 (11.44 to 18.35)	57.03 (43.10 to 69.38)	−0.91 (−1.04 to −0.78)
	Female	16.39 (10.4 to 27.86)	44.31 (26.84 to 79.85)	10.39 (8.15 to 12.51)	40.69 (31.49 to 48.57)	−1.54 (−1.71 to −1.36)
**SDI^e^ region**						
	High	11.97 (10.52 to 15.53)	9.53 (8.37 to 12.29)	11.63 (9.24 to 12.97)	12.47 (9.76 to 14.00)	−0.05 (−0.32 to 0.23)
	High-middle	21.17 (17.74 to 29.91)	22.52 (18.89 to 32.25)	15.39 (12.24 to 18.54)	23.62 (18.89 to 28.37)	−1.12 (−1.36 to −0.87)
	Middle	25.58 (19.32 to 41.28)	42.59 (30.42 to 74.27)	15.81 (12.12 to 19.05)	34.62 (26.93 to 42.53)	−1.52 (−1.66 to −1.39)
	Low-middle	14.05 (8.24 to 19.74)	16.07 (7.82 to 26.8)	11.51 (8.27 to 13.44)	17.90 (13.17 to 21.06)	−0.60 (−0.77 to −0.42)
	Low	12.08 (6.69 to 18.73)	7.38 (2.70 to 14.98)	8.78 (6.37 to 12.81)	9.06 (6.48 to 12.81)	−1.10 (−1.19 to −1.02)
**GBD^f^ region**						
	High-income Asia Pacific	14.81 (12.35 to 18.98)	2.34 (2.00 to 3.05)	9.54 (8.26 to 12.05)	1.94 (1.53 to 2.27)	−1.76 (−1.87 to −1.65)
	High-income North America	11.02 (9.18 to 15.42)	2.94 (2.44 to 4.13)	14.60 (10.72 to 16.65)	4.93 (3.57 to 5.67)	1.28 (0.78 to 1.79)
	Western Europe	8.40 (6.42 to 11.05)	3.40 (2.63 to 4.41)	8.74 (5.71 to 10.29)	5.47 (3.40 to 6.65)	0.31 (−0.18 to 0.80)
	Australasia	18.11 (15.31 to 24.14)	0.36 (0.31 to 0.48)	13.34 (11.1 to 18.06)	0.42 (0.35 to 0.58)	−1.29 (−1.71 to −0.87)
	Southern Latin America	11.36 (8.69 to 14.82)	0.55 (0.42 to 0.73)	7.91 (6.47 to 10.93)	0.55 (0.45 to 0.74)	−1.47 (−1.55 to −1.38)
	Andean Latin America	7.98 (4.06 to 11.17)	0.27 (0.12 to 0.42)	3.49 (2.62 to 4.65)	0.21 (0.16 to 0.28)	−3.16 (−3.36 to −2.96)
	Tropical Latin America	12.35 (9.06 to 17.42)	1.87 (1.32 to 2.64)	11.02 (8.83 to 16.26)	2.26 (1.84 to 3.33)	−0.40 (−0.65 to −0.14)
	Central Latin America	4.89 (3.79 to 5.92)	0.84 (0.64 to 1.05)	5.25 (3.92 to 6.77)	1.25 (0.95 to 1.61)	0.21 (0.07 to 0.34)
	Caribbean	18.76 (9.05 to 45.08)	0.70 (0.31 to 1.82)	17.09 (9.31 to 33.57)	0.76 (0.44 to 1.43)	−0.16 (−0.27 to −0.05)
	Eastern Europe	11.3 (9.31 to 16.17)	2.51 (2.03 to 3.75)	16.52 (12.98 to 22.05)	4.05 (3.00 to 5.09)	0.93 (0.76 to 1.10)
	Central Europe	27.56 (18.86 to 33.05)	3.30 (2.27 to 4.00)	25.68 (17.61 to 31.76)	4.28 (2.86 to 5.38)	−0.83 (−1.10 to −0.57)
	Central Asia	17.71 (14.63 to 24.7)	1.09 (0.91 to 1.49)	26.7 (20.54 to 41.35)	2.43 (1.83 to 3.87)	2.05 (1.29 to 2.82)
	North Africa and Middle East	25.72 (15.08 to 54.1)	10.98 (5.78 to 26.03)	15.28 (10.67 to 27.02)	8.70 (5.99 to 15.50)	−1.64 (−1.70 to −1.57)
	South Asia	7.16 (5.05 to 9.45)	6.64 (4.10 to 9.52)	6.64 (5.18 to 8.62)	10.84 (8.53 to 14.01)	−0.27 (−0.32 to −0.23)
	Southeast Asia	14.57 (9.79 to 25.00)	6.32 (3.76 to 13.05)	10.58 (8.19 to 15.14)	6.36 (4.88 to 9.60)	−1.10 (−1.29 to −0.92)
	East Asia	41.66 (31.81 to 66.61)	46.68 (34.49 to 76.35)	25.17 (16.67 to 29.43)	35.17 (22.47 to 41.52)	−1.57 (−1.87 to −1.26)
	Oceania	15.75 (7.73 to 26.27)	0.12 (0.05 to 0.22)	17.74 (8.38 to 29.45)	0.27 (0.12 to 0.47)	0.60 (0.50 to 0.70)
	Western Sub-Saharan Africa	10.69 (5.12 to 16.89)	1.64 (0.66 to 2.84)	7.59 (5.48 to 9.94)	2.90 (1.95 to 4.16)	−1.44 (−1.63 to −1.24)
	Eastern Sub-Saharan Africa	14.14 (6.39 to 24.17)	3.90 (1.19 to 8.13)	7.47 (3.34 to 14.01)	3.21 (1.44 to 5.62)	−2.19 (−2.32 to −2.07)
	Central Sub-Saharan Africa	15.22 (6.86 to 31.79)	1.01 (0.26 to 2.88)	9.20 (4.57 to 17.6)	1.03 (0.56 to 2.00)	−1.85 (−1.91 to −1.78)
	Southern Sub-Saharan Africa	12.81 (7.84 to 19.51)	0.66 (0.36 to 1.14)	9.18 (6.84 to 13.08)	0.69 (0.51 to 0.99)	−1.26 (−1.38 to −1.13)

^a^EAPC: estimated annual percentage change.

^b^ASDR: age-standardized disability-adjusted life year rate.

^c^UI: uncertainty interval.

^d^DALYs: disability-adjusted life years.

^e^SDI: sociodemographic index.

^f^GBD: Global Burden of Disease Study.

### Variation in the Myocarditis Burden at Regional and National Levels

The ASIR of myocarditis was always the highest in high and high-middle SDI regions from 1990 to 2019: 18.72 and 16.58 per 100,000 person-years in 1990, and 17.75 and 16.01 per 100,000 person-years in 2019, respectively (Table 1). Low SDI regions had the lowest ASIR of 15.29 per 100,000 person-years in 2019. The ASIR slightly decreased in all SDI regions between 1990 and 2019 (Table 1). The leading ASMR in 2019 was observed in middle SDI regions at 0.59/100,000, and high-middle SDI regions ranked second at 0.56/100,000. Low SDI regions had the lowest ASMR of 0.24/100,000 in 2019. The largest increase in the ASMR was observed in low-middle SDI regions, followed by high SDI regions. On the other hand, the other 3 regions presented downward trends in the ASMR (lowest EAPC of −0.97 in low SDI regions) (Table 2). The highest ASDR in 2019 was observed in middle SDI regions, and the lowest was observed in low SDI regions. The ASDRs in 5 SDI regions all dropped from 1990 to 2019, with the highest decrease in middle SDI regions (EAPC=−1.52) (Table 3).

With respect to 21 GBD regions, high-income Asia Pacific, high-income North America, and Eastern Europe were the top 3 regions with the highest ASIRs in 2019 (range from 17.74/100,000 to 20.07/100,000). On the contrary, the North Africa and Middle East, Andean Latin America, and Caribbean GBD regions had the lowest ASIRs in 2019 (range from 12.05/100,000 to 14.19/100,000) (Table 1). The ASIR of myocarditis changed slightly or remained stable across all GBD regions from 1990 to 2019 (Table 1). The largest 3 increases in the ASMR were observed in Central Asia, high-income North America, and Western Europe. On the contrary, the largest 3 decreases in the ASMR were observed in Andean Latin America, Western Sub-Saharan Africa, and Eastern Sub-Saharan Africa (Table 2). The changing trends in the ASMR were different across all GBD regions (Table 2). The highest 3 ASDRs in 2019 appeared in Central Asia, Central Europe, and East Asia (range from 25.17 to 26.70 per 100,000 person-years). On the contrary, the lowest 3 ASDRs in 2019 appeared in Andean Latin America, Central Latin America, and South Asia (range from 3.49 to 6.64 per 100,000 person-years) (Table 3). There were significant regional differences in the trends of ASDRs across all the GBD regions, with the most obvious increase in Central Asia (EAPC=2.05) and the largest decrease in Andean Latin America (EAPC=−3.16) (Table 3).

**Figure 2:**
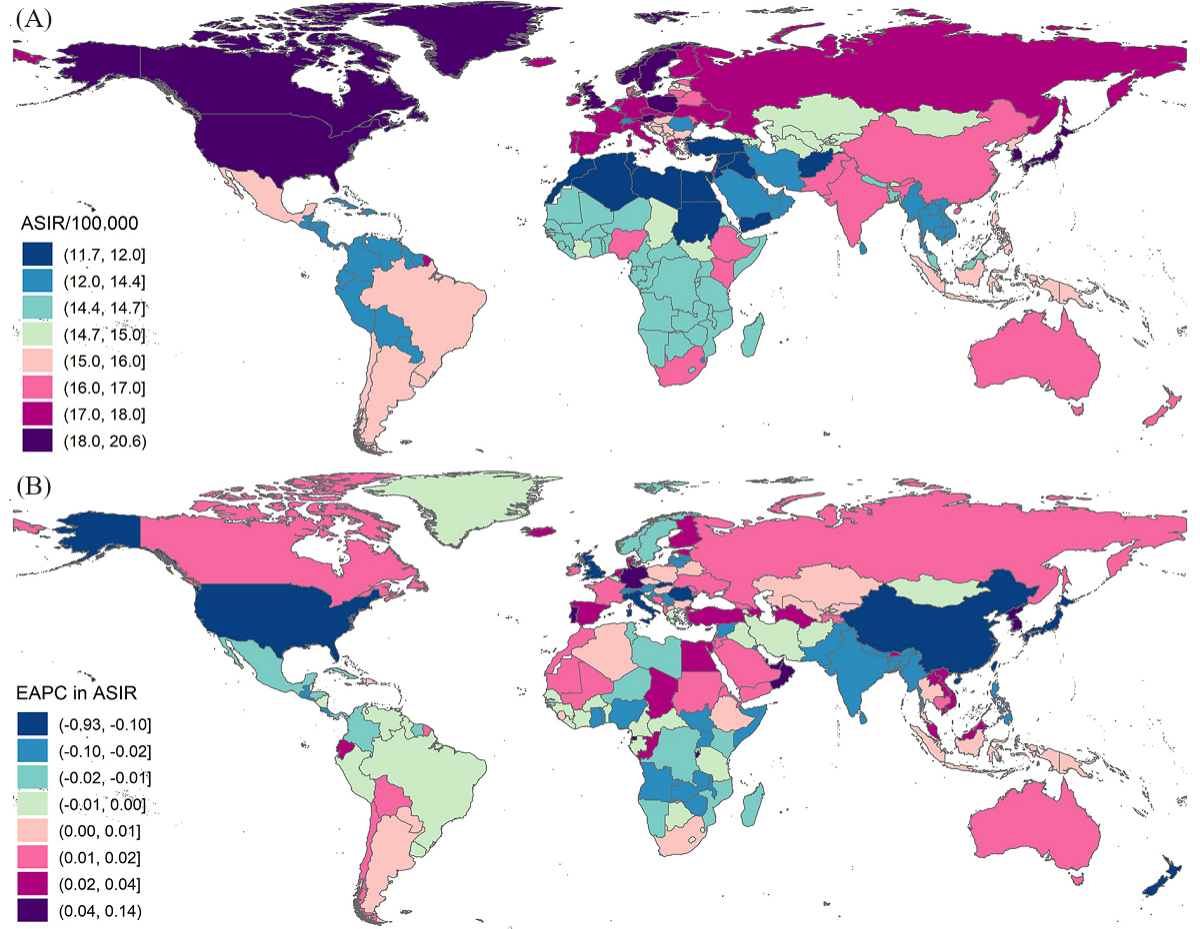
Maps of the global incidence and temporal trends of myocarditis in 204 countries and territories. (A) ASIR of myocarditis around the world in 2019; (B) EAPC in the ASIR from 1990 to 2019. ASIR: age-standardized incidence rate; EAPC: estimated annual percentage change.

**Figure 3:**
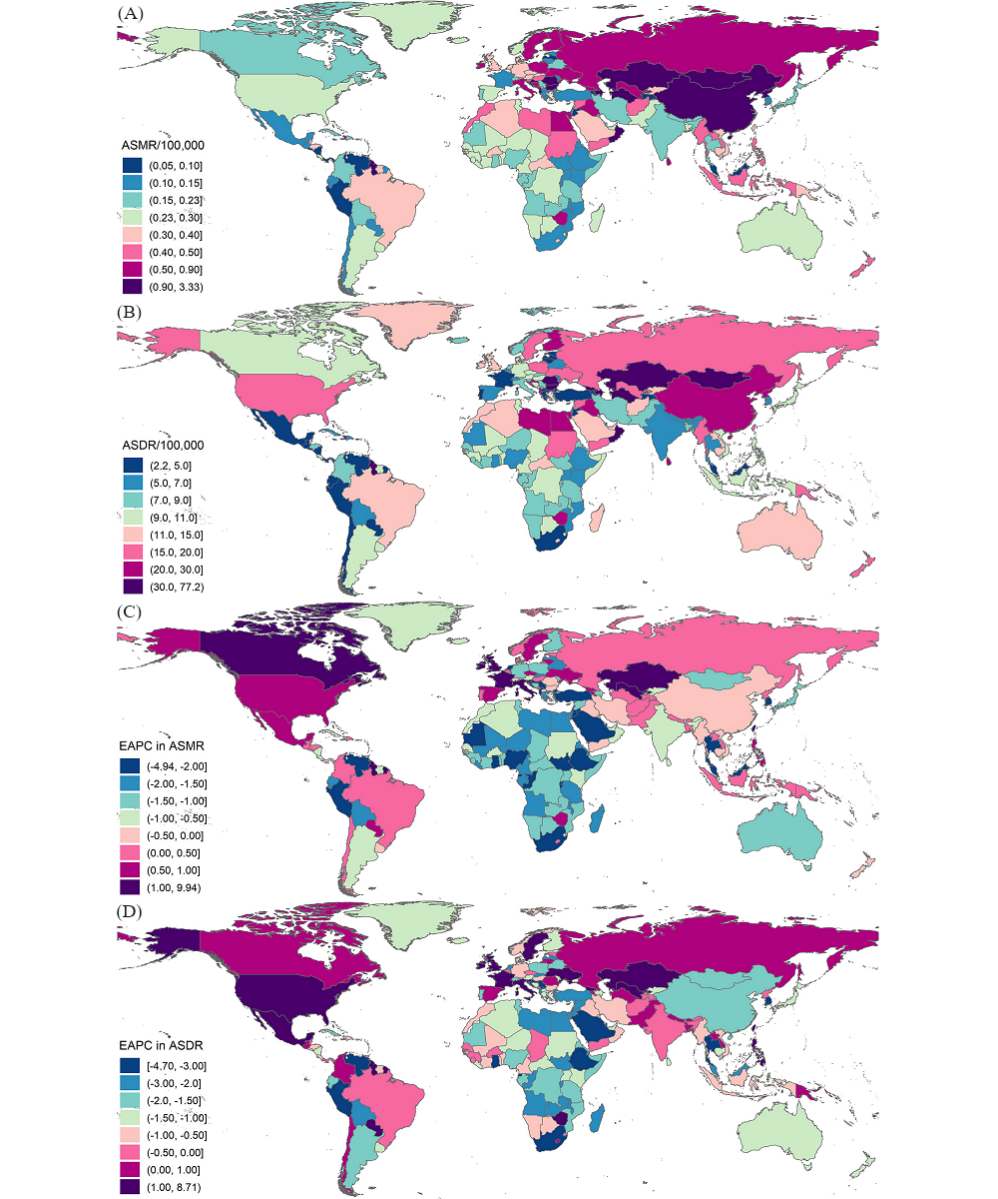
Maps of the global mortality and DALYs along with temporal trends of myocarditis in 204 countries and territories. (A) ASMR of myocarditis around the world in 2019; (B) EAPC in the ASMR from 1990 to 2019; (C) ASDR of myocarditis around the world in 2019; (D) EAPC in the ASDR from 1990 to 2019. ASDR: age-standardized DALY rate; ASMR: age-standardized mortality rate; DALYs: disability-adjusted life years; EAPC: estimated annual percentage change.

In 2019, there were 3 countries with an ASIR exceeding 20/100,000, including Austria, Japan, and Sweden, while Lebanon had the lowest ASIR (Multimedia Appendix 1; Figure 2). The difference in the ASMR of myocarditis was nearly 60 times across the world in 2019, with Romania showing the highest value (3.32/100,000) and Tajikistan showing the lowest value (0.05/100,000) (Multimedia Appendix 1; Figure 3). Similarly, the difference in the ASDR of myocarditis was nearly 30 times across the world in 2019, with Romania showing the highest value and Tajikistan showing the lowest value (Multimedia Appendix 1; Figure 3). From 1990 to 2019, 100 out of 204 countries and territories showed a rising ASIR. The largest annual increment in the ASIR was noted in Qatar, with an EAPC of 0.13. On the contrary, the fastest decline in the ASIR was noted in the United States, with an EAPC of −0.93 (Multimedia Appendix 2; Figure 2). The EAPC in the ASMR was the highest in Kazakhstan (EAPC=9.93) and the lowest in Ghana (EAPC=−4.94) from 1990 to 2019 (Multimedia Appendix 2; Figure 3). The EAPC in the ASDR was the highest in Kazakhstan (EAPC=8.70) and the lowest in Serbia (EAPC=−4.70) from 1990 to 2019 (Multimedia Appendix 2; Figure 3).

### Variation in the Myocarditis Burden in Both Genders and 5-Year Age Groups

From 1990 to 2019, the ASIR of myocarditis among men was higher than that among women (19.06/100,000 vs 13.01/100,000 in 2019) (Table 1). Similar to the finding of the ASIR, the ASMR and ASDR of myocarditis were higher among men than among women. Over the past 3 decades, all ASRs among both sexes had downward trends, except for the ASMR among males, which showed a stable trend (EAPC=0.07) (Table 2). Markedly, the decrease in the ASDR from 1990 to 2019 was greater among females (EAPC=−1.54) than among males (EAPC=−0.91) at the global level (Table 3).

**Figure 4:**
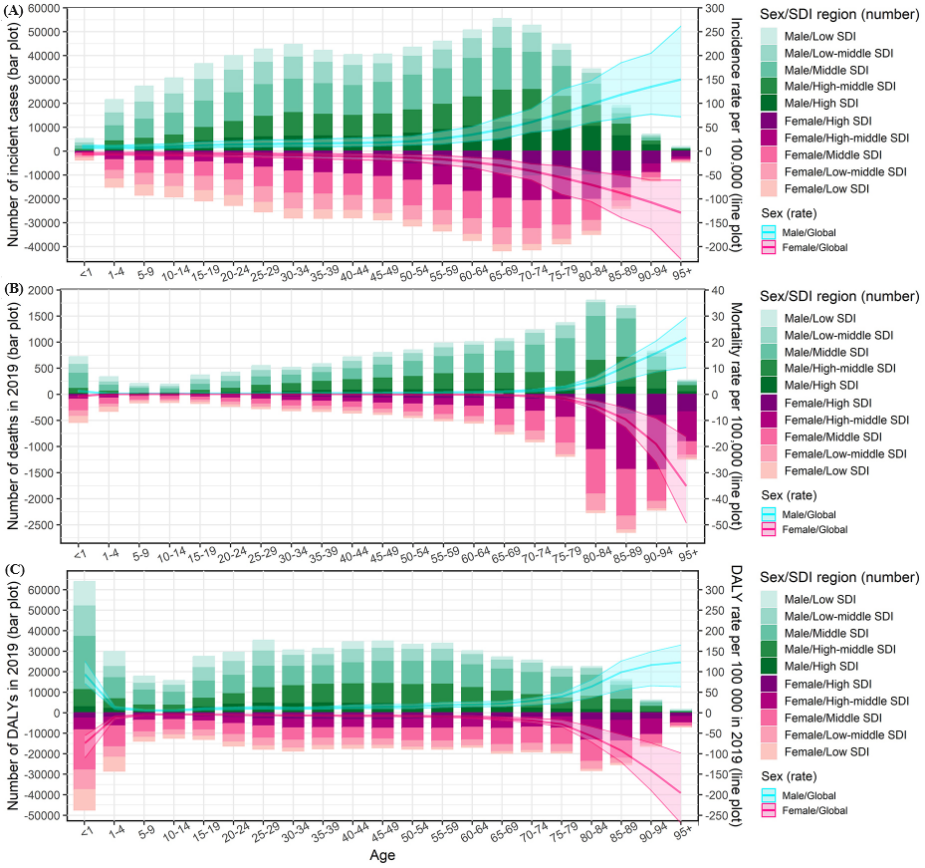
Age-standardized counts and rates of the myocarditis burden by sex/SDI region in 2019. (A) Incidence; (B) Mortality; (C) DALYs. DALYs: disability-adjusted life years; SDI: sociodemographic index.

In 2019, the incident cases displayed a bimodal distribution with age and peaked the highest in the 65-69 age group. A relatively lower peak was shown in the 30-34 age group with incident cases (Figure 4). The number of deaths peaked in the 80-84 age group among males and in the 85-89 age group among females. The under 1 age group among both sexes had the largest number of deaths younger than 20 years (Figure 4). The number of DALYs in 2019 peaked in the under 1 age group among both sexes (Figure 4).

The incidence and mortality rates per 100,000 person-years all showed approximately increasing trends with age among both sexes in 2019 (Figure 4). The DALY rate per 100,000 person-years showed a decreasing trend before the 10-14 age group and an increasing trend after the 10-14 age group with age among both sexes (Figure 4). Over the past 3 decades, the ASIRs among males and females were decreasing over all ages (EAPC<0) (Multimedia Appendix 3). From 1990 to 2019, the population older than 69 years among both sexes showed increasing trends in the ASMR and ASDR with age (EAPC>0) (Multimedia Appendix 3).

From 1990 to 2019, all SDI regions showed an increasing tendency in the absolute incident cases among the population over 30 years old (Multimedia Appendix 4). On the other hand, the ASIR showed a decreasing trend in high SDI regions among the population over 50 years old. Over the past 30 years, the ASIR in the other 4 SDI regions changed slightly across all age groups (Multimedia Appendix 4). Deaths were obviously rising in 5 SDI regions among the residents over 40 years old, especially in high-middle and middle SDI regions. Conversely, the number of deaths was decreasing in all SDI regions among the residents younger than 4 years, especially in high-middle, middle, and low-middle SDI regions (Multimedia Appendix 5). From 1990 to 2019, the ASMR in all SDI regions was steadily rising across all age groups over 85 years, except in low SDI regions where there was a decrease (Multimedia Appendix 5). The number of DALYs was decreasing in all SDI regions among the population younger than 14 years, especially in high-middle, middle, and low-middle SDI regions (Multimedia Appendix 6). The ASDR showed a similar trend as the ASMR in 5 SDI regions across all age groups over 85 years. The ASDR was obviously decreasing in the under 1 age group in all SDI regions over the past 30 years (Multimedia Appendix 6).

The EAPCs in myocarditis ASIRs varied largely among age groups in the 5 SDI regions during the past 30 years. The highest EAPC in the ASIR in high SDI regions was noted between the ages of 14 and 19 years. In the other 4 SDI regions, the highest EAPC in the ASIR was noted in the age group of older than 95 years (Multimedia Appendix 4). Globally, the EAPCs in the ASMR and ASDR showed rising trends with age, and similar trends could be found in low-middle, middle, and high-middle SDI regions. The highest EAPCs in the ASDR and ASMR in high SDI regions were noted between the ages of 44 and 54 years. The EAPCs in the ASMR and ASDR in low SDI regions were always negative at all ages (Multimedia Appendices 5 and 6).

### Potential Factors of Changing Trends

We found negative correlations between the EAPC and the ASIR (ρ=−0.15; *P=*.03), ASMR (ρ=−0.23; *P<*.001), and ASDR (ρ=−0.28; *P<*.001) in 1990 (Multimedia Appendices 7-9). On the other hand, there were no correlations between the EAPCs in the ASIR, ASMR, and ASDR and the SDI (Multimedia Appendices 7-9). The analysis of the annual ASIR of myocarditis from 1990 to 2019 and the SDI across all 21 GBD regions indicated that the ASIR in most GBD regions increased or decreased slightly, except for high-income North America that showed an obviously increasing trend first, a substantial decrease next, and an increase again (Multimedia Appendix 7). The ASMR and ASDR in Central Asia, Central Europe, and Southeast Asia changed significantly, while that in the other GBD regions changed slightly (Multimedia Appendices 8 and 9).

## Discussion

### Principal Findings

The study estimated the global burden of myocarditis from 1990 to 2019 systematically and comprehensively, which indicated the progressive and prominent influence on public health due to myocarditis. Compared with 1990, the numbers of incident cases and deaths of myocarditis both increased by above 1.6 times across the world, while the global DALYs decreased. All ASRs showed declining trends with negative EAPCs over the past 30 years. The myocarditis burden will certainly increase because of the increasing number of cases and deaths. Myocarditis is a kind of myocardial inflammatory disease diagnosed by established criteria [15,24,25]. The clinical manifestations of severe myocarditis are often fulminant, with sudden onset, extensive myocardial damage, and accompaniment by fatal arrhythmia, contributing to a high risk of sudden death [26-30]. The burden of myocarditis is a public health problem of widespread concern, particularly under the background of SARS-CoV-2 infection and mRNA vaccines [31-33]. Since the pandemic, many epidemiological studies have concluded that SARS-CoV-2 increased the incidence of myocarditis. Studies also showed that the use of novel mRNA platforms led to a higher number of reported cases than with previous platforms. Hence, fundamental data on the burden of myocarditis is helpful for investigating the burden induced by SARS-CoV-2 [34,35]. Analyses based on the differences in sex, age, year, SDI quintiles, GBD regions, and nations depicted the heterogeneities of myocarditis burden. Health policymakers should take notice of the significant implications on public health related to myocarditis globally.

This research found that higher SDI regions always showed a more significant ASIR from 1990 to 2019, which was not analyzed in a previous study of the GBD 2017 [8]. The high incidence of myocarditis in high and high-middle SDI regions may be related to better health care infrastructure and medical resources, population aging, the introduction of highly sensitive troponin and CMRI examinations, and extensive use of ICIs and vaccines [36]. Middle SDI regions accounted for the largest ASMR and ASDR in 2019, and low-middle SDI regions had the largest increase in the ASMR. The main reasons for high mortality and disability in middle SDI regions and the fastest increase in low-middle SDI regions may be delayed diagnosis, imperfect health care systems, and limited medical resources. Ventricular assist devices or extracorporeal membrane oxygenation (ECMO) need to be urgently applied in patients with cardiogenic shock or severe ventricular dysfunction due to myocarditis [37,38]. However, mechanical support devices have not been widely applied in middle and low-middle SDI regions. We also found that all ASRs in low SDI regions were the lowest, which may be due to insufficient health care infrastructure and resources. Definitely, the medical health care system and quality of data sources need to be improved in lower SDI regions for reducing the burden of myocarditis.

At the GBD level, high-income Asia Pacific, high-income North America, and East Asia accounted for the top 3 highest ASIRs in 2019. At the national level in 2019, the ASIR in Austria, Japan, and Sweden exceeded 20/100,000. The EAPCs in the ASMR and ASDR were the highest in Kazakhstan in the past 3 decades. Furthermore, there were significant negative correlations between the EAPCs in ASRs and the baseline ASRs in our results, which demonstrated that countries and territories with higher ASIRs, ASMRs, and ASDRs in 1990 went through more rapid decreases or slow increases in the ASRs of myocarditis from 1990 to 2019. Policymakers of countries and territories with higher ASRs were more likely to attach importance to the prevention of myocarditis and facilitate more reasonable policy formulation and resource allocation. In 2019, there were significant regional differences in the trends of ASRs, which provided accumulating evidence for the epidemiological transition of myocarditis across all GBD regions, nations, and territories. Policymakers should facilitate country-specific health research to allocate limited medical resources more reasonably based on the national information on the myocarditis disease burden.

The ASRs of myocarditis were higher among males than among females from 1990 to 2019 globally. Over the past 3 decades, all ASRs among both sexes showed downward trends, except for the ASMR among males, which showed a stable trend, and females had a more significant decrease in the ASDR than males at the global level. Previous studies have already displayed a slightly higher incidence rate of myocarditis in men [8,29]. More recently, mRNA-based COVID-19 vaccines were reported to be responsible for an increased risk of myocarditis on the basis of passive surveillance reporting in the United States, especially among adolescent boys and young men after the second vaccine dose [39]. Men were more likely to experience a severe myocarditis developmental trajectory. Differences in innate immunity between men and women may contribute to a different prognosis. Testosterone and estrogen play important roles in the immune response. Testosterone can increase the affinity of the virus to myocytes and trigger a T helper type 1 immune response [40], inhibit the quantity of anti-inflammatory cells, and upregulate cardiac fibrotic remodeling genes [41]. Men with myocarditis have been found to have higher levels of heart failure biomarkers, creatine kinase, myoglobin, and T helper 17–associated cytokines [42]. On the other hand, women can develop a better regulatory immune response. Estrogen can promote the differentiation of T cells to T helper 2 cells and reduce the T helper type 1 immune response [43].

For estimating the age characteristics of the burden of myocarditis more accurately, we adopted a more refined age grouping scheme than that in the previous study of the GBD 2017 [8]. We found that the highest peak of deaths occurred in the under 1 age group among those aged younger than 20 years, and the number of DALYs in 2019 peaked in the under 1 age group among both sexes. There were 2 peaks in the incidence of myocarditis in childhood, and a high incidence was found in infancy and adolescence, with infants having poor prognoses [44]. Infantile myocarditis was more prone to causing severe ventricular dysfunction, which led to a higher probability of requiring mechanical circulatory support compared with that among patients of other ages. Myocarditis was proved to have caused 9% of cases of sudden infant death syndrome on autopsy [6]. It is noteworthy that infants are more susceptible to enteroviruses characterized by direct myocardial injury. The virus can trigger a cascade of hyperinflammatory responses and induce more robust immune responses among adolescents and young adults, especially among males. Previous research indicated that children and young adults had a higher incidence of myocarditis and mortality attributed to myocarditis compared with middle-aged and older adults [8,45]. However, our data showed that the incidence and mortality rates had increasing trends with age, and the DALY rate had a decreasing trend before the 10-14 age group and an increasing trend after the 10-14 age group with age in 2019. Differences in etiology, pathogenesis, and diagnosis strategies may be important reasons for the differences in the burden of myocarditis among children, adults, and elderly people. Senior citizens had high ASRs and numbers of cases in 2019, which demonstrated that elderly people gradually predominated in terms of the burden of myocarditis. The burden of myocarditis shifted to elderly people mainly due to population aging, ICI therapy, hypoimmunity, and more concomitant underlying diseases. Policymakers should be aware of the high DALYs in infants and the shifting of the burden to elderly people.

### Limitations

This study has several limitations. First, the study may be prone to sampling bias. The GBD model of myocarditis was reconstructed based on a large number of varying quality, sparse, or limited scope data sources. This can affect the accuracy and applicability of the findings across all regions and populations. The possibility of missed cases of myocarditis, which are either undiagnosed or unreported, could also lead to an underestimation of the actual disease burden. Therefore, the estimation of the burden of myocarditis may deviate from the actuality to some extent, especially in some underdeveloped regions with scarce prior information. Second, longitudinal data were lacking in this study. The absence of data extending beyond the timeframe limits the understanding of recent trends and the ability to evaluate the effect of newer developments in the diagnosis, treatment, and prevention of myocarditis, especially after SARS-CoV-2, with some studies reporting its relation to an increased risk of myocarditis [34]. Third, the classification of myocarditis was not available in this study. There are many causes of myocarditis, among which viruses are the most common. Multiple types of viruses, including enteroviruses, adenoviruses, erythroparvoviruses, viruses from the *Herpesviridae* family, and SARS-CoV-2, can lead to myocarditis. Other etiologies include autoimmunity, vaccines, ICIs, and exposure to toxins and drugs. The clinical course of myocarditis caused by different factors may be different, and further investigations are necessary. Finally, the Dallas criteria in 1987 for myocarditis considered endomyocardial biopsy to be the standard diagnosis [46,47]. However, the diagnostic strategy has changed with the introduction of highly sensitive troponin and CMRI examinations in the past 20 years. In routine clinical practice, it is often sufficient to establish the diagnosis of myocarditis with the combination of symptoms and signs, laboratory examinations, and imaging studies [3]. It is unclear if the diagnostic criteria of myocarditis were kept the same among different areas. Further investigations are needed to evaluate the impact of changes and regional differences in the diagnostic workup for the assessment of myocarditis burden.

### Conclusions

In general, myocarditis remains an important cause of early death and chronic disability, and it negatively impacted the global disease burden from 1990 to 2019. The numbers of incident cases and deaths associated with myocarditis have increased significantly. On the other hand, the ASRs of myocarditis showed decreasing trends from 1990 to 2019. High SDI regions showed more significant ASIRs, while middle SDI regions showed the highest ASMRs and ASDRs in 2019. Males consistently showed higher ASRs of myocarditis than females from 1990 to 2019 globally. Senior citizens had high incident cases and deaths among both sexes in 2019. Peak numbers of DALYs for both sexes were noted in the under 1 age group in 2019. At the national level, the EAPCs in ASRs had significant negative correlations with the baseline ASRs in 1990. Policymakers should develop targeted control strategies based on gender, region, age, and SDI; strengthen aging-related health research; and take notice of the changes in the epidemic characteristics of myocarditis. Targeted control strategies should be developed to reduce the high DALYs in infants and the increasing burden of myocarditis in the elderly population based on the diversity of myocarditis burden explicated in our study.

## Appendix

Multimedia Appendix 1 The incident cases, deaths, disability-adjusted life years, and corresponding burden rate of myocarditis in 204 countries and territories in 2019.

Multimedia Appendix 2 Estimated annual percentage change in the burden rate of myocarditis in 204 countries and territories from 1990 to 2019.

Multimedia Appendix 3 EAPC in the age-standardized rates of the myocarditis burden across all age groups by sex in 2019. (A) Incidence; (B) Mortality; (C) DALYs. DALYs: disability-adjusted life years; EAPC: estimated annual percentage change.

Multimedia Appendix 4 Changes in the incidence of myocarditis across all age groups in global and SDI regions from 1990 to 2019. (A) Number of incident cases; (B) Age-standardized incidence rate; (C) EAPC in the age-standardized incidence rate. EAPC: estimated annual percentage change; SDI: sociodemographic index.

Multimedia Appendix 5 Changes in the mortality of myocarditis across all age groups in global and SDI regions from 1990 to 2019. (A) Number of deaths; (B) Age-standardized mortality rate; (C) EAPC in the age-standardized mortality rate. EAPC: estimated annual percentage change; SDI: sociodemographic index.

Multimedia Appendix 6 Changes in the DALYs of myocarditis across all age groups in global and SDI regions from 1990 to 2019. (A) Number of DALYs; (B) Age-standardized DALY rate; (C) EAPC in the age-standardized DALY rate. DALYs: disability-adjusted life years; EAPC: estimated annual percentage change; SDI: sociodemographic index.

Multimedia Appendix 7 Factors affecting the EAPC in the ASIR of myocarditis and the changing trends of the ASIR across all GBD regions from 1990 to 2019. The size of the circle indicates the number of incident cases of myocarditis. (A) Correlation between the EAPC in the ASIR and the corresponding ASIR in 1990; (B) Correlation between the EAPC in the ASIR and the SDI in 2019; (C) Changing trends of the ASIR across all GBD regions from 1990 to 2019. ASIR: age-standardized incidence rate; EAPC: estimated annual percentage change; GBD: Global Burden of Disease Study; SDI: sociodemographic index.

Multimedia Appendix 8 Factors affecting the EAPC in the ASMR of myocarditis and the changing trends of the ASMR across all GBD regions from 1990 to 2019. The size of the circle indicates the number of deaths associated with myocarditis. (A) Correlation between the EAPC in the ASMR and the corresponding ASMR in 1990; (B) Correlation between the EAPC in the ASMR and the SDI in 2019; (C) Changing trends of the ASIR across all GBD regions from 1990 to 2019. ASMR: age-standardized mortality rate; EAPC: estimated annual percentage change; GBD: Global Burden of Disease Study; SDI: sociodemographic index.

Multimedia Appendix 9 Factors affecting the EAPC in the ASDR of myocarditis and the changing trends of the ASDR across all GBD regions from 1990 to 2019. The size of the circle indicates the number of DALYs of myocarditis. (A) Correlation between the EAPC in the ASDR and the corresponding ASDR in 1990; (B) Correlation between the EAPC in the ASDR and the SDI in 2019; (C) Changing trends of the ASDR across all GBD regions from 1990 to 2019. ASDR: age-standardized DALY rate; DALYs: disability-adjusted life years; EAPC: estimated annual percentage change; GBD: Global Burden of Disease Study; SDI: sociodemographic index.

## Abbreviations

ASDR: age-standardized disability-adjusted life year rate
ASIR: age-standardized incidence rate
ASMR: age-standardized mortality rate
ASR: age-standardized rate
CMRI: cardiac magnetic resonance imaging
DALYs: disability-adjusted life years
EAPC: estimated annual percentage change
GBD: Global Burden of Disease Study
ICD: International Classification of Diseases
ICI: immune checkpoint inhibitor
SDI: sociodemographic index
UI: uncertainty interval

## Data Availability

The Global Burden of Disease Study 2019 is a publicly available database [48], and we fully complied with data usage requirements.

## References

[1] Mensah GA, Roth GA, Fuster V (2019). The Global Burden of Cardiovascular Diseases and Risk Factors: 2020 and Beyond. J Am Coll Cardiol.

[2] Roth GA, Mensah GA, Johnson CO, Addolorato G, Ammirati E, Baddour LM, Barengo NC, Beaton AZ, Benjamin EJ, Benziger CP, Bonny A, Brauer M, Brodmann M, Cahill TJ, Carapetis J, Catapano AL, Chugh SS, Cooper LT, Coresh J, Criqui M, DeCleene N, Eagle KA, Emmons-Bell S, Feigin VL, Fernández-Solà J, Fowkes G, Gakidou E, Grundy SM, He FJ, Howard G, Hu F, Inker L, Karthikeyan G, Kassebaum N, Koroshetz W, Lavie C, Lloyd-Jones D, Lu HS, Mirijello A, Temesgen AM, Mokdad A, Moran AE, Muntner P, Narula J, Neal B, Ntsekhe M, Moraes de Oliveira G, Otto C, Owolabi M, Pratt M, Rajagopalan S, Reitsma M, Ribeiro ALP, Rigotti N, Rodgers A, Sable C, Shakil S, Sliwa-Hahnle K, Stark B, Sundström J, Timpel P, Tleyjeh IM, Valgimigli M, Vos T, Whelton PK, Yacoub M, Zuhlke L, Murray C, Fuster V, GBD-NHLBI-JACC Global Burden of Cardiovascular Diseases Writing Group (2020). Global Burden of Cardiovascular Diseases and Risk Factors, 1990-2019: Update From the GBD 2019 Study. J Am Coll Cardiol.

[3] Basso C (2022). Myocarditis. N Engl J Med.

[4] Maron BJ, Doerer JJ, Haas TS, Tierney DM, Mueller FO (2009). Sudden deaths in young competitive athletes: analysis of 1866 deaths in the United States, 1980-2006. Circulation.

[5] Shatz A, Hiss J, Arensburg B (1997). Myocarditis misdiagnosed as sudden infant death syndrome (SIDS). Med Sci Law.

[6] Rajs J, Hammarquist F (1988). Sudden infant death in Stockholm. A forensic pathology study covering ten years. Acta Paediatr Scand.

[7] Råsten-Almqvist P, Eksborg S, Rajs J (2002). Myocarditis and sudden infant death syndrome. APMIS.

[8] Wang X, Bu X, Wei L, Liu J, Yang D, Mann DL, Ma A, Hayashi T (2021). Global, Regional, and National Burden of Myocarditis From 1990 to 2017: A Systematic Analysis Based on the Global Burden of Disease Study 2017. Front Cardiovasc Med.

[9] Charpentier S, Beaune S, Joly LM, Khoury A, Duchateau F, Briot R, Renaud B, Ageron F, Network IRU (2018). Management of chest pain in the French emergency healthcare system: the prospective observational EPIDOULTHO study. Eur J Emerg Med.

[10] Rizzo S, Carturan E, De Gaspari M, Pilichou K, Thiene G, Basso C (2019). Update on cardiomyopathies and sudden cardiac death. Forensic Sci Res.

[11] Mahmood SS, Fradley MG, Cohen JV, Nohria A, Reynolds KL, Heinzerling LM, Sullivan RJ, Damrongwatanasuk R, Chen CL, Gupta D, Kirchberger MC, Awadalla M, Hassan MZO, Moslehi JJ, Shah SP, Ganatra S, Thavendiranathan P, Lawrence DP, Groarke JD, Neilan TG (2018). Myocarditis in Patients Treated With Immune Checkpoint Inhibitors. J Am Coll Cardiol.

[12] Ammirati E, Lupi L, Palazzini M, Hendren NS, Grodin JL, Cannistraci CV, Schmidt M, Hekimian G, Peretto G, Bochaton T, Hayek A, Piriou N, Leonardi S, Guida S, Turco A, Sala S, Uribarri A, Van de Heyning CM, Mapelli M, Campodonico J, Pedrotti P, Barrionuevo Sánchez M, Ariza Sole A, Marini M, Matassini MV, Vourc'h M, Cannatà A, Bromage DI, Briguglia D, Salamanca J, Diez-Villanueva P, Lehtonen J, Huang F, Russel S, Soriano F, Turrini F, Cipriani M, Bramerio M, Di Pasquale M, Grosu A, Senni M, Farina D, Agostoni P, Rizzo S, De Gaspari M, Marzo F, Duran JM, Adler ED, Giannattasio C, Basso C, McDonagh T, Kerneis M, Combes A, Camici PG, de Lemos JA, Metra M (2022). Prevalence, Characteristics, and Outcomes of COVID-19-Associated Acute Myocarditis. Circulation.

[13] Bemtgen X, Kaier K, Rilinger J, Rottmann F, Supady A, von Zur Mühlen C, Westermann D, Wengenmayer T, Staudacher DL (2022). Myocarditis mortality with and without COVID-19: insights from a national registry. Clin Res Cardiol.

[14] Dagan N, Barda N, Kepten E, Miron O, Perchik S, Katz MA, Hernán M, Lipsitch M, Reis B, Balicer RD (2021). BNT162b2 mRNA Covid-19 Vaccine in a Nationwide Mass Vaccination Setting. N Engl J Med.

[15] Baritussio A, Giordani AS, Rizzo S, Masiero G, Iliceto S, Marcolongo R, Caforio AL (2022). Management of myocarditis in clinical practice. Minerva Cardiol Angiol.

[16] Ferreira VM, Schulz-Menger J, Holmvang G, Kramer CM, Carbone I, Sechtem U, Kindermann I, Gutberlet M, Cooper LT, Liu P, Friedrich MG (2018). Cardiovascular Magnetic Resonance in Nonischemic Myocardial Inflammation: Expert Recommendations. J Am Coll Cardiol.

[17] Pahuja M, Adegbala O, Mishra T, Akintoye E, Chehab O, Mony S, Singh M, Ando T, Abubaker H, Yassin A, Subahi A, Shokr M, Ranka S, Briasoulis A, Kapur NK, Burkhoff D, Afonso L (2019). Trends in the Incidence of In-Hospital Mortality, Cardiogenic Shock, and Utilization of Mechanical Circulatory Support Devices in Myocarditis (Analysis of National Inpatient Sample Data, 2005-2014). J Card Fail.

[18] GBD 2019 Risk Factors Collaborators (2020). Global burden of 87 risk factors in 204 countries and territories, 1990-2019: a systematic analysis for the Global Burden of Disease Study 2019. Lancet.

[19] GBD 2019 DiseasesInjuries Collaborators (2020). Global burden of 369 diseases and injuries in 204 countries and territories, 1990-2019: a systematic analysis for the Global Burden of Disease Study 2019. Lancet.

[20] Liu X, Cao Y, Wang W (2023). Burden of and Trends in Urticaria Globally, Regionally, and Nationally from 1990 to 2019: Systematic Analysis. JMIR Public Health Surveill.

[21] Global Burden of Disease Study 2019 (GBD 2019) Code. Institute for Health Metrics and Evaluation | Global Health Data Exchange.

[22] Stevens GA, Alkema L, Black RE, Boerma JT, Collins GS, Ezzati M, Grove JT, Hogan DR, Hogan MC, Horton R, Lawn JE, Marušić A, Mathers CD, Murray CJL, Rudan I, Salomon JA, Simpson PJ, Vos T, Welch V, (The GATHER Working Group) (2016). Guidelines for Accurate and Transparent Health Estimates Reporting: the GATHER statement. Lancet.

[23] Yang X, Man J, Chen H, Zhang T, Yin X, He Q, Lu M (2021). Temporal trends of the lung cancer mortality attributable to smoking from 1990 to 2017: A global, regional and national analysis. Lung Cancer.

[24] Cipriani M, Merlo M, Gabrielli D, Ammirati E, Autore C, Basso C, Caforio A, Caldarola P, Camici P, Di Lenarda A, Frustaci A, Imazio M, Oliva F, Pedrotti P, Perazzolo Marra M, Rapezzi C, Urbinati S, Zecchin M, Filardi PP, Colivicchi F, Indolfi C, Frigerio M, Sinagra G (2020). [ANMCO/SIC Consensus document on the management of myocarditis]. G Ital Cardiol (Rome).

[25] Dec GW, Palacios IF, Fallon JT, Aretz HT, Mills J, Lee DC, Johnson RA (1985). Active myocarditis in the spectrum of acute dilated cardiomyopathies. Clinical features, histologic correlates, and clinical outcome. N Engl J Med.

[26] Ammirati E, Veronese G, Bottiroli M, Wang DW, Cipriani M, Garascia A, Pedrotti P, Adler ED, Frigerio M (2021). Update on acute myocarditis. Trends Cardiovasc Med.

[27] Ammirati E, Veronese G, Cipriani M, Moroni F, Garascia A, Brambatti M, Adler ED, Frigerio M (2018). Acute and Fulminant Myocarditis: a Pragmatic Clinical Approach to Diagnosis and Treatment. Curr Cardiol Rep.

[28] Peretto G, Sala S, De Luca G, Marcolongo R, Campochiaro C, Sartorelli S, Tresoldi M, Foppoli L, Palmisano A, Esposito A, De Cobelli F, Rizzo S, Thiene G, Basso C, Dagna L, Caforio ALP, Della Bella P (2020). Immunosuppressive Therapy and Risk Stratification of Patients With Myocarditis Presenting With Ventricular Arrhythmias. JACC Clin Electrophysiol.

[29] Fabre A, Sheppard MN (2006). Sudden adult death syndrome and other non-ischaemic causes of sudden cardiac death. Heart.

[30] Felker GM, Hu W, Hare JM, Hruban RH, Baughman KL, Kasper EK (1999). The spectrum of dilated cardiomyopathy. The Johns Hopkins experience with 1,278 patients. Medicine (Baltimore).

[31] Castiello T, Georgiopoulos G, Finocchiaro G, Claudia M, Gianatti A, Delialis D, Aimo A, Prasad S (2022). COVID-19 and myocarditis: a systematic review and overview of current challenges. Heart Fail Rev.

[32] Rahman A (2022). Myocarditis and related complications of SARS-CoV-2 infection. Aust J Gen Pract.

[33] Ilyin SO (2021). A Recursive Model of the Spread of COVID-19: Modelling Study. JMIR Public Health Surveill.

[34] Fairweather D, Beetler DJ, Di Florio DN, Musigk N, Heidecker B, Cooper LT (2023). COVID-19, Myocarditis and Pericarditis. Circ Res.

[35] Abu Mouch S, Roguin A, Hellou E, Ishai A, Shoshan U, Mahamid L, Zoabi M, Aisman M, Goldschmid N, Berar Yanay N (2021). Myocarditis following COVID-19 mRNA vaccination. Vaccine.

[36] Sexson Tejtel SK, Munoz FM, Al-Ammouri I, Savorgnan F, Guggilla RK, Khuri-Bulos N, Phillips L, Engler RJM (2022). Myocarditis and pericarditis: Case definition and guidelines for data collection, analysis, and presentation of immunization safety data. Vaccine.

[37] Ammirati E, Veronese G, Brambatti M, Merlo M, Cipriani M, Potena L, Sormani P, Aoki T, Sugimura K, Sawamura A, Okumura T, Pinney S, Hong K, Shah P, Van de Heyning CM, Montero S, Petrella D, Huang F, Schmidt M, Raineri C, Lala A, Varrenti M, Foà A, Leone O, Gentile P, Artico J, Agostini V, Patel R, Garascia A, Van Craenenbroeck EM, Hirose K, Isotani A, Murohara T, Arita Y, Sionis A, Fabris E, Hashem S, Garcia-Hernando V, Oliva F, Greenberg B, Shimokawa H, Sinagra G, Adler ED, Frigerio M, Camici PG, Braun (2019). Fulminant Versus Acute Nonfulminant Myocarditis in Patients With Left Ventricular Systolic Dysfunction. J Am Coll Cardiol.

[38] Kociol RD, Cooper LT, Fang JC, Moslehi JJ, Pang PS, Sabe MA, Shah RV, Sims DB, Thiene G, Vardeny O, American Heart Association Heart FailureTransplantation Committee of the Council on Clinical Cardiology (2020). Recognition and Initial Management of Fulminant Myocarditis: A Scientific Statement From the American Heart Association. Circulation.

[39] Witberg G, Barda N, Hoss S, Richter I, Wiessman M, Aviv Y, Grinberg T, Auster O, Dagan N, Balicer RD, Kornowski R (2021). Myocarditis after Covid-19 Vaccination in a Large Health Care Organization. N Engl J Med.

[40] Huber SA, Pfaeffle B (1994). Differential Th1 and Th2 cell responses in male and female BALB/c mice infected with coxsackievirus group B type 3. J Virol.

[41] Barcena ML, Jeuthe S, Niehues MH, Pozdniakova S, Haritonow N, Kühl A, Messroghli DR, Regitz-Zagrosek V (2021). Sex-Specific Differences of the Inflammatory State in Experimental Autoimmune Myocarditis. Front Immunol.

[42] Fairweather D, Beetler DJ, Musigk N, Heidecker B, Lyle MA, Cooper LT, Bruno KA (2023). Sex and gender differences in myocarditis and dilated cardiomyopathy: An update. Front Cardiovasc Med.

[43] Fairweather D, Cooper LT, Blauwet LA (2013). Sex and gender differences in myocarditis and dilated cardiomyopathy. Curr Probl Cardiol.

[44] Butts RJ, Boyle GJ, Deshpande SR, Gambetta K, Knecht KR, Prada-Ruiz CA, Richmond ME, West SC, Lal AK (2017). Characteristics of Clinically Diagnosed Pediatric Myocarditis in a Contemporary Multi-Center Cohort. Pediatr Cardiol.

[45] Li L, Zhang Y, Burke A, Xue A, Zhao Z, Fowler D, Shen Y, Li L (2017). Demographic, clinical and pathological features of sudden deaths due to myocarditis: Results from a state-wide population-based autopsy study. Forensic Sci Int.

[46] Aretz HT (1987). Myocarditis: the Dallas criteria. Hum Pathol.

[47] Seferović PM, Tsutsui H, McNamara DM, Ristić AD, Basso C, Bozkurt B, Cooper LT, Filippatos G, Ide T, Inomata T, Klingel K, Linhart A, Lyon AR, Mehra MR, Polovina M, Milinković I, Nakamura K, Anker SD, Veljić I, Ohtani T, Okumura T, Thum T, Tschöpe C, Rosano G, Coats AJS, Starling RC (2021). Heart Failure Association of the ESC, Heart Failure Society of America and Japanese Heart Failure Society Position statement on endomyocardial biopsy. Eur J Heart Fail.

[48] Global Burden of Disease Study 2019 (GBD 2019) Data Resources. Institute for Health Metrics and Evaluation | Global Health Data Exchange.

