# Supplementary material for: Global, Regional, and National Burden of Myocarditis in 204 Countries and Territories From 1990 to 2019: Updated Systematic Analysis
Source: JMIR Public Health Surveill. 2024 Jan 11;10:e46635. doi: 10.2196/46635 (PMC10811576; doi:10.2196/46635)
Supplement: Multimedia Appendix 1 [file publichealth_v10i1e46635_app1.docx]

| **Multimedia Appendix 1. The incident cases, deaths, disability-adjusted life years, and corresponding burden rate of myocarditis in 204 countries and territories in 2019.** | | | | | | |
| --- | --- | --- | --- | --- | --- | --- |
| **Location** | **Incident cases**  **(95%UI)** | **ASIR/100 000**  **(95%UI)** | **Deaths**  **(95%UI)** | **ASMR/100 000**  **(95%UI)** | **DALYs**  **(95%UI)** | **ASDR/100 000**  **(95%UI)** |
| Afghanistan | 3093 (2421-3892) | 11.77 (9.5-14.36) | 79 (41-145) | 0.47 (0.2-1.02) | 3889 (2174-6962) | 13.93 (7.41-25.69) |
| Albania | 520 (416-630) | 15.95 (12.82-19.43) | 37 (20-79) | 1.03 (0.59-2.2) | 846 (505-1757) | 29.24 (17.87-56.85) |
| Algeria | 4496 (3573-5536) | 11.82 (9.54-14.41) | 115 (65-287) | 0.35 (0.2-0.87) | 4984 (2821-11787) | 12.59 (7.2-30.18) |
| American Samoa | 8 (6-9) | 15.2 (12.24-18.36) | 0 (0-0) | 0.14 (0.1-0.2) | 4 (3-6) | 7.34 (5.14-10.7) |
| Andorra | 19 (15-23) | 17.35 (14.04-21.12) | 0 (0-1) | 0.32 (0.21-0.46) | 10 (7-14) | 9.03 (6.53-12.49) |
| Angola | 3001 (2356-3725) | 14.49 (11.66-17.68) | 40 (23-86) | 0.22 (0.1-0.46) | 2365 (1354-5055) | 8.77 (4.97-18.31) |
| Antigua and Barbuda | 13 (11-16) | 14.17 (11.46-17.15) | 1 (1-1) | 0.98 (0.75-1.26) | 23 (18-30) | 25.34 (19.81-33.12) |
| Argentina | 7636 (6207-9305) | 15.35 (12.44-18.65) | 153 (115-196) | 0.29 (0.23-0.38) | 4260 (3467-5844) | 9.16 (7.4-12.9) |
| Armenia | 513 (410-621) | 14.87 (11.87-17.99) | 3 (2-6) | 0.1 (0.06-0.15) | 104 (62-187) | 3.15 (2.04-5.27) |
| Australia | 5263 (4203-6399) | 16.44 (13.19-19.94) | 94 (73-135) | 0.28 (0.22-0.41) | 3321 (2606-4760) | 12.38 (9.65-17.68) |
| Austria | 2587 (2076-3162) | 20.58 (17.02-24.84) | 56 (42-82) | 0.32 (0.25-0.44) | 1164 (902-1508) | 9.53 (6.88-12.26) |
| Azerbaijan | 1451 (1142-1782) | 14.9 (11.89-18.03) | 114 (51-308) | 1.24 (0.63-2.73) | 4354 (1945-12398) | 40.35 (19.56-105.7) |
| Bahamas | 54 (43-66) | 14.11 (11.42-17.07) | 1 (1-1) | 0.27 (0.19-0.36) | 37 (25-49) | 9.55 (6.56-12.69) |
| Bahrain | 160 (121-205) | 12.07 (9.73-14.69) | 1 (0-2) | 0.07 (0.05-0.12) | 43 (24-88) | 2.97 (1.97-5.31) |
| Bangladesh | 21360 (16929-26137) | 14.69 (11.81-17.95) | 340 (151-573) | 0.28 (0.12-0.46) | 12608 (6037-20425) | 8.51 (4.16-13.79) |
| Barbados | 55 (44-68) | 14.14 (11.44-17.1) | 1 (1-2) | 0.33 (0.24-0.43) | 35 (25-45) | 9.71 (7.18-12.76) |
| Belarus | 1907 (1521-2329) | 16.11 (12.96-19.46) | 24 (14-43) | 0.18 (0.1-0.32) | 728 (368-1374) | 6.58 (3.33-12.46) |
| Belgium | 2365 (1874-2906) | 14.61 (11.75-17.75) | 70 (37-107) | 0.26 (0.16-0.37) | 1072 (743-1364) | 6.13 (4.62-7.44) |
| Belize | 49 (39-60) | 14.29 (11.57-17.3) | 1 (1-2) | 0.37 (0.28-0.53) | 47 (37-64) | 12.72 (9.99-17.37) |
| Benin | 1287 (1010-1600) | 14.55 (11.7-17.75) | 18 (11-26) | 0.27 (0.17-0.41) | 984 (551-1530) | 9.05 (5.77-13.19) |
| Bermuda | 13 (11-17) | 14.14 (11.43-17.1) | 0 (0-0) | 0.25 (0.19-0.35) | 7 (5-9) | 7.33 (5.36-10.25) |
| Bhutan | 99 (78-122) | 14.75 (11.84-18.02) | 1 (1-2) | 0.23 (0.13-0.37) | 43 (25-70) | 6.47 (3.87-10.51) |
| Bolivia | 1445 (1157-1766) | 14.09 (11.37-17.2) | 18 (10-27) | 0.23 (0.13-0.35) | 575 (341-833) | 5.59 (3.34-7.94) |
| Bosnia and Herzegovina | 661 (526-807) | 15.85 (12.75-19.34) | 28 (11-50) | 0.58 (0.24-1.01) | 454 (228-693) | 10.03 (5.11-14.68) |
| Botswana | 276 (217-343) | 14.46 (11.62-17.63) | 4 (2-8) | 0.24 (0.12-0.51) | 213 (104-424) | 9.86 (4.96-19.58) |
| Brazil | 35864 (28947-43757) | 15.76 (12.71-19.2) | 659 (532-999) | 0.31 (0.25-0.47) | 22279 (18190-32974) | 11.25 (9.01-16.72) |
| Brunei | 74 (59-93) | 19.08 (15.43-23.09) | 2 (2-3) | 0.61 (0.42-0.79) | 120 (87-153) | 26.6 (19.2-33.54) |
| Bulgaria | 1516 (1193-1874) | 15.88 (12.77-19.37) | 186 (117-315) | 1.51 (0.98-2.47) | 3603 (2431-5823) | 37.54 (25.99-58.32) |
| Burkina Faso | 2315 (1824-2874) | 14.51 (11.67-17.7) | 34 (20-54) | 0.3 (0.19-0.45) | 1788 (976-3517) | 9.39 (6-13.9) |
| Burundi | 1211 (950-1503) | 14.68 (11.79-17.93) | 15 (4-31) | 0.15 (0.04-0.33) | 1026 (335-2169) | 7.64 (2.78-15.06) |
| Cambodia | 2063 (1630-2522) | 14.21 (11.41-17.16) | 41 (29-59) | 0.38 (0.22-0.54) | 1860 (1147-3164) | 12.38 (8.23-19.29) |
| Cameroon | 3043 (2390-3767) | 14.6 (11.74-17.82) | 42 (24-63) | 0.28 (0.17-0.42) | 2222 (1231-3503) | 9.22 (5.57-13.5) |
| Canada | 9771 (7744-12029) | 18.96 (15.36-23.1) | 89 (50-118) | 0.19 (0.11-0.24) | 3798 (2529-4729) | 10.33 (7.18-12.52) |
| Cape Verde | 74 (59-90) | 14.55 (11.69-17.74) | 1 (1-2) | 0.26 (0.17-0.4) | 48 (34-67) | 8.99 (6.46-12.3) |
| Central African Republic | 541 (425-668) | 14.46 (11.62-17.63) | 11 (5-24) | 0.31 (0.14-0.6) | 628 (244-1541) | 12.37 (6.23-26.43) |
| Chad | 1599 (1255-1989) | 14.72 (11.85-17.99) | 22 (13-34) | 0.27 (0.16-0.42) | 1244 (681-2115) | 9.06 (5.81-13.87) |
| Chile | 3253 (2608-3967) | 15.41 (12.49-18.71) | 27 (21-39) | 0.13 (0.1-0.18) | 852 (691-1130) | 4.56 (3.63-5.9) |
| China | 275060 (221842-336581) | 16.94 (13.77-20.48) | 13141 (7672-16378) | 0.92 (0.54-1.14) | 341265 (217738-404254) | 25.39 (16.8-29.68) |
| Colombia | 7186 (5818-8758) | 14.15 (11.45-17.11) | 77 (54-109) | 0.16 (0.11-0.22) | 3628 (2297-5079) | 8.15 (4.95-11.63) |
| Comoros | 88 (71-108) | 14.58 (11.71-17.79) | 1 (0-2) | 0.13 (0.04-0.31) | 45 (17-91) | 6.64 (2.63-13.56) |
| Congo | 583 (458-721) | 14.59 (11.72-17.81) | 7 (4-17) | 0.22 (0.09-0.49) | 368 (196-859) | 8.16 (4.26-18.56) |
| Cook Islands | 3 (2-4) | 15.14 (12.2-18.28) | 0 (0-0) | 0.08 (0.05-0.11) | 1 (0-1) | 3.66 (2.48-5) |
| Costa Rica | 703 (566-856) | 14.15 (11.46-17.11) | 13 (9-22) | 0.26 (0.18-0.43) | 526 (367-727) | 10.92 (7.71-14.88) |
| Croatia | 896 (709-1099) | 15.24 (12.42-18.39) | 125 (65-190) | 1.44 (0.77-2.12) | 1551 (1017-2111) | 20.35 (14.82-26.47) |
| Cuba | 2148 (1705-2654) | 14.22 (11.51-17.2) | 27 (17-48) | 0.17 (0.12-0.28) | 787 (575-1188) | 6.86 (5.28-9.44) |
| Cyprus | 269 (216-333) | 17.05 (13.9-20.85) | 4 (2-6) | 0.29 (0.13-0.44) | 95 (58-127) | 7.02 (4.15-9.4) |
| Czech | 2505 (1980-3078) | 17.69 (14.2-21.63) | 66 (50-95) | 0.36 (0.27-0.48) | 1509 (1000-1986) | 10.59 (6.33-14.45) |
| Cote d'Ivoire | 2760 (2170-3428) | 14.73 (11.82-17.99) | 33 (20-50) | 0.25 (0.15-0.37) | 1772 (1031-2890) | 8.13 (5.2-12.12) |
| Denmark | 1305 (1040-1608) | 16.15 (13.11-19.46) | 36 (27-45) | 0.33 (0.25-0.41) | 721 (538-866) | 9.13 (6.56-10.94) |
| Djibouti | 136 (107-169) | 14.78 (11.89-18.05) | 1 (1-3) | 0.14 (0.05-0.34) | 90 (36-184) | 7.32 (3.1-15.36) |
| Dominica | 11 (9-14) | 14.28 (11.53-17.29) | 1 (0-1) | 0.78 (0.56-1.09) | 16 (12-22) | 21.58 (15.85-29.23) |
| Dominican Republic | 1435 (1150-1747) | 14.24 (11.52-17.22) | 16 (7-27) | 0.19 (0.07-0.32) | 511 (316-766) | 5 (3.03-7.46) |
| DR Congo | 9050 (7091-11193) | 14.52 (11.68-17.7) | 127 (47-254) | 0.25 (0.07-0.53) | 6793 (3001-12918) | 9.28 (3.73-18.22) |
| Ecuador | 2274 (1832-2748) | 13.99 (11.36-16.81) | 16 (11-22) | 0.12 (0.08-0.16) | 530 (394-731) | 3.23 (2.41-4.39) |
| Egypt | 9753 (7711-12082) | 11.93 (9.63-14.49) | 440 (247-867) | 0.53 (0.31-1.01) | 28142 (14245-57407) | 28.34 (15.25-56.97) |
| El Salvador | 864 (701-1049) | 14.01 (11.34-16.93) | 6 (3-9) | 0.1 (0.05-0.15) | 179 (119-245) | 2.91 (1.94-4) |
| Equatorial Guinea | 145 (113-180) | 14.56 (11.7-17.75) | 1 (1-3) | 0.16 (0.07-0.33) | 66 (32-139) | 5.94 (3.12-11.85) |
| Eritrea | 695 (545-862) | 14.46 (11.62-17.62) | 8 (3-15) | 0.15 (0.05-0.29) | 500 (192-948) | 7.57 (3.1-14.13) |
| Estonia | 294 (233-361) | 16.17 (13-19.52) | 3 (2-4) | 0.12 (0.09-0.17) | 75 (50-101) | 4.65 (3.05-6.29) |
| Eswatini | 126 (100-156) | 14.38 (11.55-17.53) | 2 (1-4) | 0.27 (0.15-0.53) | 112 (62-213) | 10.62 (6.13-20.38) |
| Ethiopia | 12386 (9885-15233) | 16.4 (13.29-19.83) | 103 (32-228) | 0.13 (0.03-0.34) | 6980 (2728-12880) | 6.63 (2.68-13.84) |
| Fiji | 124 (99-151) | 15.16 (12.22-18.32) | 2 (1-3) | 0.27 (0.17-0.37) | 132 (87-186) | 14.73 (9.63-20.57) |
| Finland | 1434 (1131-1766) | 17.55 (14.24-21.15) | 62 (47-104) | 0.63 (0.5-0.84) | 1579 (1165-1900) | 21.92 (15.35-26.69) |
| France | 16172 (12941-19978) | 17.07 (13.83-20.82) | 201 (115-284) | 0.14 (0.08-0.18) | 3961 (2525-4999) | 4.81 (2.94-6.02) |
| Gabon | 205 (162-252) | 14.5 (11.66-17.68) | 2 (1-5) | 0.19 (0.1-0.42) | 111 (65-250) | 7.19 (4.35-16.04) |
| Gambia | 238 (187-295) | 14.59 (11.73-17.81) | 3 (2-5) | 0.28 (0.17-0.41) | 158 (93-244) | 8.55 (5.47-12.7) |
| Georgia | 684 (549-836) | 14.86 (11.99-18.06) | 48 (12-82) | 0.89 (0.23-1.52) | 1522 (377-2614) | 30.68 (8.58-51.92) |
| Germany | 21886 (17241-27305) | 17.1 (13.86-20.79) | 614 (467-860) | 0.35 (0.28-0.47) | 12383 (9926-15563) | 10.9 (8.55-13.07) |
| Ghana | 3518 (2779-4351) | 14.48 (11.65-17.66) | 28 (18-46) | 0.19 (0.12-0.3) | 1315 (825-2242) | 5.06 (3.5-8) |
| Greece | 2719 (2135-3400) | 17.11 (13.87-20.85) | 34 (21-48) | 0.13 (0.09-0.17) | 563 (415-728) | 3.48 (2.83-4.83) |
| Greenland | 12 (9-14) | 19.21 (15.57-23.45) | 0 (0-0) | 0.25 (0.13-0.35) | 7 (4-10) | 13.36 (7.88-19.59) |
| Grenada | 15 (12-18) | 14.2 (11.46-17.19) | 1 (0-1) | 0.68 (0.51-0.97) | 19 (15-28) | 18.93 (14.55-26.86) |
| Guam | 27 (21-32) | 15.26 (12.3-18.46) | 0 (0-1) | 0.27 (0.19-0.47) | 26 (18-42) | 15.59 (10.68-25.27) |
| Guatemala | 2043 (1633-2498) | 14.09 (11.4-17.04) | 16 (10-22) | 0.13 (0.08-0.19) | 699 (476-1115) | 4.31 (3.03-6.35) |
| Guinea | 1325 (1048-1633) | 14.61 (11.75-17.84) | 22 (13-31) | 0.3 (0.18-0.45) | 1102 (638-1707) | 10.07 (6.39-14.34) |
| Guinea-Bissau | 194 (152-240) | 14.49 (11.66-17.67) | 3 (2-4) | 0.31 (0.19-0.48) | 157 (91-251) | 10.18 (6.32-15) |
| Guyana | 97 (77-119) | 14.16 (11.45-17.13) | 13 (8-19) | 2.08 (1.25-2.92) | 517 (315-732) | 71.03 (43-99.67) |
| Haiti | 1355 (1079-1669) | 14.16 (11.47-17.14) | 79 (38-148) | 0.87 (0.48-1.47) | 4592 (1736-10652) | 36.33 (15.87-75.36) |
| Honduras | 1114 (891-1369) | 14.17 (11.47-17.14) | 19 (10-30) | 0.37 (0.17-0.61) | 557 (332-839) | 7.9 (4.49-12.08) |
| Hungary | 2063 (1638-2538) | 15.79 (12.71-19.29) | 83 (56-134) | 0.45 (0.31-0.71) | 1421 (1035-2148) | 10.36 (7.72-15.11) |
| Iceland | 75 (61-91) | 17.27 (13.98-21.03) | 2 (1-2) | 0.26 (0.2-0.32) | 32 (26-40) | 7.56 (5.95-9.33) |
| India | 207168 (165262-253439) | 16.36 (13.26-19.84) | 2118 (1565-2808) | 0.2 (0.15-0.27) | 78165 (59778-102071) | 6.15 (4.72-8.03) |
| Indonesia | 37929 (30243-46674) | 15.99 (12.95-19.44) | 664 (464-959) | 0.41 (0.24-0.56) | 22378 (15422-36359) | 10.32 (7.55-15.25) |
| Iran | 10580 (8388-13053) | 13.17 (10.57-16.07) | 167 (107-222) | 0.23 (0.15-0.31) | 7022 (5077-9563) | 8.94 (6.53-12.45) |
| Iraq | 3981 (3133-4977) | 11.77 (9.49-14.33) | 214 (159-287) | 0.87 (0.59-1.16) | 9816 (7132-13756) | 27.58 (20.59-36.28) |
| Ireland | 1021 (823-1245) | 17.15 (13.9-20.88) | 43 (15-68) | 0.59 (0.21-0.93) | 791 (349-1147) | 12.82 (5.87-18.26) |
| Israel | 1746 (1418-2119) | 17.13 (13.88-20.88) | 15 (10-27) | 0.14 (0.1-0.22) | 488 (385-655) | 5.02 (3.83-6.54) |
| Italy | 16296 (12951-20056) | 17.83 (14.66-21.33) | 1187 (190-2005) | 0.59 (0.13-0.97) | 12069 (4130-18154) | 8.57 (3.97-11.43) |
| Jamaica | 420 (341-510) | 14.22 (11.5-17.21) | 4 (3-6) | 0.14 (0.1-0.2) | 146 (104-203) | 5.37 (3.82-7.43) |
| Japan | 41119 (32225-51344) | 20.51 (16.75-24.61) | 664 (340-899) | 0.23 (0.16-0.27) | 14900 (11292-17442) | 10.22 (8.61-13.93) |
| Jordan | 1123 (883-1401) | 11.9 (9.6-14.49) | 8 (6-10) | 0.1 (0.07-0.14) | 388 (289-503) | 3.72 (2.82-4.87) |
| Kazakhstan | 2611 (2072-3171) | 14.84 (11.86-17.95) | 197 (100-365) | 1.15 (0.54-1.95) | 7219 (4100-14006) | 38 (21-72.04) |
| Kenya | 6002 (4769-7388) | 16.25 (13.15-19.72) | 39 (14-86) | 0.11 (0.04-0.27) | 2285 (1028-4547) | 5.19 (2.33-10.66) |
| Kiribati | 14 (11-18) | 14.99 (12.07-18.09) | 1 (0-1) | 0.6 (0.35-0.85) | 35 (18-55) | 29.14 (15.74-45.14) |
| Kuwait | 462 (356-588) | 11.99 (9.67-14.56) | 7 (5-9) | 0.18 (0.14-0.25) | 360 (277-473) | 8.16 (6.38-10.71) |
| Kyrgyzstan | 845 (672-1031) | 14.87 (11.87-17.99) | 20 (10-29) | 0.35 (0.19-0.49) | 911 (432-1326) | 14.42 (7.03-20.81) |
| Laos | 858 (680-1058) | 14.37 (11.54-17.4) | 19 (12-29) | 0.44 (0.26-0.65) | 858 (471-1637) | 13.46 (8.44-23.16) |
| Latvia | 420 (334-519) | 15.6 (12.59-18.93) | 2 (2-4) | 0.08 (0.06-0.12) | 73 (55-100) | 3.43 (2.32-4.55) |
| Lebanon | 611 (491-743) | 11.7 (9.44-14.24) | 14 (6-37) | 0.28 (0.12-0.72) | 545 (244-1379) | 10.54 (4.69-26.97) |
| Lesotho | 246 (194-303) | 14.42 (11.58-17.59) | 5 (3-9) | 0.33 (0.19-0.62) | 217 (136-384) | 11.86 (7.48-21.2) |
| Liberia | 514 (405-639) | 14.7 (11.8-17.95) | 6 (3-10) | 0.25 (0.13-0.42) | 317 (176-520) | 7.97 (4.62-12.42) |
| Libya | 730 (572-913) | 11.82 (9.54-14.41) | 21 (11-47) | 0.42 (0.21-0.97) | 1224 (625-2781) | 24.42 (11.48-56.61) |
| Lithuania | 658 (520-814) | 16.77 (13.52-20.37) | 5 (4-8) | 0.12 (0.09-0.17) | 160 (116-217) | 4.98 (3.36-6.59) |
| Luxembourg | 141 (113-171) | 18.01 (14.6-21.73) | 5 (4-7) | 0.48 (0.34-0.64) | 97 (73-125) | 11.61 (8.83-15.1) |
| Madagascar | 2776 (2175-3422) | 14.62 (11.75-17.83) | 51 (18-109) | 0.23 (0.07-0.51) | 3319 (1237-6914) | 12.09 (4.58-24.87) |
| Malawi | 1892 (1476-2348) | 14.47 (11.63-17.65) | 19 (6-41) | 0.13 (0.04-0.33) | 1270 (472-2516) | 7.02 (2.68-14.45) |
| Malaysia | 4219 (3351-5142) | 14.49 (11.65-17.54) | 25 (17-36) | 0.09 (0.06-0.14) | 1360 (877-1897) | 4.61 (2.99-6.41) |
| Maldives | 64 (50-80) | 14.84 (11.96-17.97) | 1 (1-1) | 0.21 (0.15-0.33) | 38 (26-62) | 8.39 (5.78-12.87) |
| Mali | 2222 (1744-2755) | 14.69 (11.8-17.94) | 33 (21-53) | 0.3 (0.18-0.47) | 1807 (1047-3103) | 9.45 (6.07-14.56) |
| Malta | 109 (87-135) | 17.21 (13.95-20.98) | 10 (7-12) | 1.12 (0.88-1.42) | 185 (150-231) | 30.83 (24.99-39.32) |
| Marshall Islands | 7 (6-9) | 15.34 (12.37-18.52) | 0 (0-0) | 0.43 (0.19-0.66) | 11 (5-17) | 20.05 (9.27-30.56) |
| Mauritania | 448 (354-551) | 14.65 (11.77-17.9) | 5 (3-8) | 0.22 (0.13-0.34) | 215 (109-379) | 6.66 (3.64-11.27) |
| Mauritius | 206 (163-252) | 14.31 (11.49-17.32) | 1 (1-2) | 0.08 (0.06-0.12) | 42 (31-63) | 3.07 (2.27-4.59) |
| Mexico | 19131 (15383-23374) | 15.81 (12.75-19.26) | 153 (90-197) | 0.14 (0.08-0.19) | 5603 (4185-7460) | 4.85 (3.58-6.37) |
| Micronesia | 13 (10-16) | 15.17 (12.23-18.34) | 0 (0-1) | 0.4 (0.15-0.62) | 18 (7-29) | 18.15 (6.89-28.61) |
| Moldova | 723 (576-886) | 16.17 (13-19.51) | 6 (4-8) | 0.12 (0.09-0.18) | 207 (154-274) | 5.06 (3.77-6.73) |
| Monaco | 10 (8-13) | 17.13 (13.88-20.85) | 0 (0-0) | 0.27 (0.18-0.38) | 4 (3-6) | 7.92 (5.72-10.72) |
| Mongolia | 435 (345-539) | 14.87 (11.87-17.99) | 44 (24-63) | 1.83 (0.79-2.72) | 1652 (1109-2305) | 52.41 (32.81-72.38) |
| Montenegro | 117 (93-142) | 15.89 (12.77-19.38) | 6 (3-11) | 0.79 (0.32-1.35) | 111 (60-170) | 14.09 (8.29-20.89) |
| Morocco | 3948 (3132-4863) | 11.77 (9.5-14.33) | 114 (61-277) | 0.41 (0.21-1.04) | 4360 (2432-10172) | 13.47 (7.37-32.37) |
| Mozambique | 2936 (2303-3644) | 14.44 (11.61-17.6) | 31 (10-64) | 0.13 (0.04-0.32) | 2177 (770-4372) | 6.87 (2.61-13.67) |
| Myanmar | 7155 (5645-8704) | 14.21 (11.41-17.19) | 177 (115-267) | 0.43 (0.25-0.63) | 7888 (4309-14896) | 15.89 (9.08-29.66) |
| Namibia | 280 (223-344) | 14.45 (11.62-17.62) | 4 (2-8) | 0.24 (0.13-0.48) | 188 (101-381) | 8.7 (4.84-17.18) |
| Nauru | 1 (1-1) | 15.18 (12.22-18.28) | 0 (0-0) | 0.39 (0.14-0.83) | 2 (1-5) | 20.63 (7.7-44.23) |
| Nepal | 3824 (3029-4669) | 14.55 (11.7-17.77) | 43 (22-67) | 0.21 (0.11-0.34) | 1453 (818-2195) | 5.66 (3.16-8.64) |
| Netherlands | 4098 (3268-5063) | 17.18 (13.92-20.93) | 114 (80-151) | 0.33 (0.24-0.43) | 1807 (1382-2585) | 7.19 (5.32-10.99) |
| New Zealand | 958 (787-1152) | 16.55 (13.81-19.62) | 24 (18-29) | 0.41 (0.3-0.49) | 887 (637-1037) | 18.64 (13.42-21.68) |
| Nicaragua | 771 (618-944) | 14.12 (11.42-17.07) | 3 (2-5) | 0.08 (0.05-0.11) | 158 (111-208) | 2.7 (1.87-3.53) |
| Niger | 2237 (1753-2788) | 14.62 (11.75-17.85) | 28 (15-46) | 0.28 (0.15-0.46) | 1659 (850-2867) | 8.72 (5.02-14.05) |
| Nigeria | 24696 (19691-30243) | 16.26 (13.19-19.7) | 226 (148-330) | 0.21 (0.15-0.29) | 11933 (7440-18506) | 6.71 (4.64-9.55) |
| Niue | 0 (0-0) | 15.15 (12.2-18.32) | 0 (0-0) | 0.26 (0.13-0.44) | 0 (0-0) | 13.3 (7.06-22.78) |
| North Korea | 4194 (3366-5124) | 15.08 (12.19-18.26) | 232 (130-362) | 0.94 (0.5-1.49) | 6320 (4147-9087) | 24.99 (16.46-36.01) |
| North Macedonia | 396 (313-482) | 15.99 (12.85-19.5) | 2 (1-4) | 0.12 (0.07-0.17) | 92 (52-137) | 4.44 (2.54-6.55) |
| Northern Mariana Islands | 7 (5-8) | 15.31 (12.34-18.5) | 0 (0-0) | 0.25 (0.16-0.5) | 5 (3-10) | 12 (7.38-23.58) |
| Norway | 1339 (1066-1655) | 18.96 (15.21-23.26) | 29 (22-37) | 0.28 (0.22-0.35) | 577 (448-673) | 8.27 (6.21-9.49) |
| Oman | 448 (340-580) | 12.12 (9.76-14.73) | 25 (15-33) | 1.04 (0.65-1.36) | 1364 (794-1865) | 37.87 (22.59-50.41) |
| Pakistan | 27535 (21892-33780) | 16.42 (13.31-19.86) | 331 (234-457) | 0.26 (0.19-0.35) | 16102 (10184-24746) | 8.32 (5.81-11.58) |
| Palau | 3 (2-3) | 15.42 (12.43-18.65) | 0 (0-0) | 0.25 (0.17-0.34) | 2 (1-3) | 11.66 (7.89-15.83) |
| Palestine | 447 (350-558) | 11.73 (9.46-14.26) | 5 (2-8) | 0.21 (0.07-0.33) | 241 (147-324) | 6.19 (3.17-8.65) |
| Panama | 594 (480-720) | 14.25 (11.53-17.24) | 4 (3-5) | 0.09 (0.06-0.12) | 155 (108-215) | 3.77 (2.61-5.26) |
| Papua New Guinea | 1172 (937-1449) | 15.34 (12.35-18.55) | 31 (13-55) | 0.34 (0.17-0.55) | 2130 (783-3998) | 18.23 (7.71-31.87) |
| Paraguay | 892 (718-1087) | 14.23 (11.5-17.21) | 7 (4-10) | 0.12 (0.08-0.18) | 282 (181-396) | 4.32 (2.84-6.07) |
| Peru | 4698 (3803-5721) | 14.11 (11.4-17.22) | 33 (21-52) | 0.1 (0.06-0.15) | 1015 (702-1532) | 3.02 (2.1-4.53) |
| Philippines | 15267 (12156-18723) | 15.72 (12.64-19.11) | 258 (112-358) | 0.46 (0.12-0.7) | 8344 (6443-11379) | 9.31 (5.85-11.52) |
| Poland | 8992 (7139-11015) | 18.16 (14.54-22.07) | 459 (295-799) | 0.67 (0.44-1.14) | 8196 (5697-13405) | 15.3 (11.14-24) |
| Portugal | 2882 (2285-3553) | 17.97 (14.64-21.74) | 40 (23-56) | 0.15 (0.1-0.2) | 650 (444-805) | 3.96 (2.87-4.79) |
| Puerto Rico | 742 (585-922) | 14.12 (11.42-17.07) | 6 (4-12) | 0.11 (0.07-0.19) | 164 (110-277) | 4.38 (3.04-7.05) |
| Qatar | 298 (225-391) | 12.65 (10.21-15.38) | 2 (1-4) | 0.15 (0.09-0.22) | 136 (77-208) | 5.94 (3.43-8.54) |
| Romania | 3625 (2868-4480) | 14 (11.2-17.04) | 1182 (565-1740) | 3.32 (1.58-4.87) | 23502 (10037-36003) | 77.2 (35.38-116.43) |
| Russia | 32025 (25499-39178) | 17.9 (14.42-21.74) | 1186 (634-1572) | 0.59 (0.35-0.76) | 30955 (21875-40070) | 18.1 (13.71-24.58) |
| Rwanda | 1375 (1079-1697) | 14.44 (11.62-17.6) | 14 (5-32) | 0.15 (0.04-0.37) | 870 (324-1722) | 7.22 (2.73-15.32) |
| Saint Kitts and Nevis | 9 (7-11) | 14.2 (11.47-17.19) | 0 (0-0) | 0.3 (0.21-0.43) | 6 (4-8) | 9.21 (6.07-13.77) |
| Saint Lucia | 28 (22-34) | 14.2 (11.47-17.18) | 2 (2-3) | 1.18 (0.88-1.51) | 59 (45-76) | 30.97 (23.52-39.52) |
| Saint Vincent and the Grenadines | 18 (14-22) | 14.32 (11.58-17.34) | 1 (0-1) | 0.53 (0.4-0.7) | 20 (15-26) | 17.3 (13.21-22.85) |
| Samoa | 28 (22-34) | 15.25 (12.28-18.42) | 0 (0-1) | 0.28 (0.15-0.41) | 26 (15-38) | 12.8 (7.35-18.81) |
| San Marino | 8 (6-10) | 17.05 (13.82-20.74) | 0 (0-1) | 0.55 (0.34-0.81) | 7 (4-10) | 13.54 (9.05-20.04) |
| Sao Tome and Principe | 23 (18-29) | 14.63 (11.75-17.85) | 0 (0-0) | 0.23 (0.15-0.35) | 13 (8-21) | 7.61 (4.93-11.27) |
| Saudi Arabia | 3590 (2766-4596) | 12.09 (9.75-14.71) | 83 (54-138) | 0.35 (0.24-0.51) | 3851 (2556-6635) | 11.89 (8.55-17.64) |
| Senegal | 1665 (1316-2045) | 14.62 (11.75-17.84) | 21 (12-32) | 0.25 (0.14-0.38) | 1032 (576-1585) | 8.11 (4.71-12.2) |
| Serbia | 1696 (1345-2080) | 15.23 (12.19-18.6) | 20 (12-44) | 0.19 (0.12-0.35) | 626 (423-890) | 7.44 (4.7-10.44) |
| Seychelles | 15 (12-18) | 14.45 (11.6-17.49) | 1 (0-1) | 0.63 (0.47-0.9) | 24 (18-35) | 23.74 (17.84-33.93) |
| Sierra Leone | 882 (696-1090) | 14.65 (11.77-17.88) | 12 (7-19) | 0.25 (0.16-0.39) | 674 (378-1160) | 8.94 (5.5-13.55) |
| Singapore | 1220 (971-1486) | 19.07 (15.48-23.06) | 40 (31-62) | 0.6 (0.47-0.9) | 1817 (1495-2464) | 30.28 (25.27-39.85) |
| Slovakia | 1155 (919-1419) | 17.17 (13.85-20.84) | 28 (16-63) | 0.35 (0.2-0.75) | 543 (380-914) | 7.84 (5.65-12.12) |
| Slovenia | 457 (362-562) | 15.85 (12.71-19.21) | 24 (11-38) | 0.47 (0.22-0.73) | 309 (170-460) | 8.73 (4.73-12.38) |
| Solomon Islands | 77 (61-95) | 15.3 (12.34-18.46) | 2 (1-4) | 0.34 (0.13-0.63) | 108 (40-211) | 16.12 (6.01-32.2) |
| Somalia | 1979 (1540-2462) | 14.49 (11.64-17.66) | 34 (8-74) | 0.18 (0.04-0.43) | 2511 (633-5762) | 9.85 (2.69-20.27) |
| South Africa | 8199 (6540-10025) | 16.15 (13.05-19.66) | 58 (43-103) | 0.13 (0.09-0.22) | 2581 (1906-4310) | 4.85 (3.66-8.02) |
| South Korea | 12401 (9908-15237) | 19.08 (15.45-23.08) | 75 (43-99) | 0.12 (0.07-0.15) | 2594 (1978-3476) | 5.36 (3.93-6.85) |
| South Sudan | 961 (754-1183) | 14.7 (11.82-17.97) | 12 (5-25) | 0.13 (0.06-0.29) | 931 (400-1949) | 7.9 (3.79-14.96) |
| Spain | 11425 (9040-14197) | 17.12 (13.87-20.88) | 302 (107-514) | 0.25 (0.11-0.39) | 4008 (2112-5637) | 5.47 (3.27-6.95) |
| Sri Lanka | 3252 (2574-3977) | 14.25 (11.44-17.25) | 183 (116-324) | 0.84 (0.52-1.55) | 6391 (4152-10732) | 28.78 (18.86-49.16) |
| Sudan | 3647 (2874-4573) | 11.85 (9.56-14.44) | 129 (61-289) | 0.45 (0.21-1.08) | 7694 (3558-16827) | 18.41 (8.79-39.65) |
| Suriname | 82 (66-101) | 14.15 (11.45-17.12) | 2 (1-2) | 0.31 (0.22-0.43) | 62 (41-84) | 10.98 (7.23-14.96) |
| Sweden | 2914 (2312-3572) | 20.13 (16.35-24.34) | 113 (85-148) | 0.58 (0.4-0.7) | 2605 (1786-3178) | 19.27 (12.42-24.06) |
| Switzerland | 1739 (1376-2145) | 13.93 (11.19-16.83) | 31 (23-47) | 0.18 (0.14-0.24) | 671 (516-823) | 5.78 (4.13-6.91) |
| Syria | 1554 (1218-1928) | 11.76 (9.47-14.33) | 47 (24-115) | 0.41 (0.21-0.99) | 2199 (1084-5089) | 16.42 (8.25-37.48) |
| Taiwan-China | 4202 (3494-5030) | 14.46 (12.26-16.84) | 131 (71-189) | 0.4 (0.22-0.57) | 4122 (2375-5832) | 15.46 (9.16-21.42) |
| Tajikistan | 1129 (880-1394) | 14.91 (11.9-18.05) | 4 (3-5) | 0.05 (0.04-0.07) | 199 (147-275) | 2.25 (1.69-3.06) |
| Tanzania | 5957 (4709-7358) | 14.56 (11.71-17.76) | 81 (30-158) | 0.15 (0.05-0.35) | 5790 (2314-11161) | 8.55 (3.56-16.25) |
| Thailand | 11736 (9352-14410) | 14.32 (11.49-17.34) | 143 (95-238) | 0.17 (0.12-0.27) | 4952 (3599-7515) | 6.94 (5.26-9.96) |
| Timor-Leste | 160 (127-196) | 14.43 (11.58-17.46) | 3 (2-5) | 0.41 (0.23-0.61) | 137 (79-246) | 11.45 (7.39-18.16) |
| Togo | 849 (669-1049) | 14.44 (11.6-17.59) | 11 (7-16) | 0.27 (0.16-0.41) | 555 (347-833) | 8.64 (5.72-12.58) |
| Tokelau | 0 (0-0) | 15.31 (12.32-18.51) | 0 (0-0) | 0.26 (0.14-0.4) | 0 (0-0) | 12.59 (6.99-19.24) |
| Tonga | 14 (11-17) | 15.12 (12.17-18.28) | 0 (0-0) | 0.13 (0.07-0.21) | 7 (3-11) | 6.37 (3.45-10.6) |
| Trinidad and Tobago | 229 (183-282) | 14.23 (11.5-17.21) | 9 (6-12) | 0.56 (0.37-0.76) | 294 (191-409) | 20.64 (13.74-28.43) |
| Tunisia | 1405 (1119-1721) | 11.74 (9.47-14.3) | 34 (17-94) | 0.3 (0.15-0.81) | 1204 (615-3292) | 10.56 (5.3-29.03) |
| Turkey | 10021 (7953-12302) | 11.74 (9.47-14.29) | 101 (71-154) | 0.13 (0.09-0.19) | 3452 (2568-4550) | 4.75 (3.33-6.24) |
| Turkmenistan | 679 (539-830) | 14.95 (11.93-18.09) | 69 (43-121) | 1.5 (0.96-2.58) | 3032 (1981-5028) | 60.3 (39.99-99.76) |
| Tuvalu | 2 (1-2) | 15.26 (12.3-18.42) | 0 (0-0) | 0.34 (0.18-0.53) | 2 (1-3) | 16.25 (8.91-24.39) |
| Uganda | 4046 (3153-5032) | 14.47 (11.64-17.65) | 42 (15-77) | 0.13 (0.04-0.28) | 2917 (1155-5365) | 6.51 (2.64-11.88) |
| UK | 16886 (13514-20719) | 18.6 (14.99-22.73) | 365 (234-436) | 0.33 (0.2-0.4) | 9086 (5427-11098) | 11.66 (6.73-14.41) |
| Ukraine | 10030 (7993-12281) | 17.9 (14.42-21.74) | 338 (187-486) | 0.55 (0.32-0.76) | 8322 (6007-11135) | 16.23 (12.14-22.28) |
| United Arab Emirates | 985 (722-1329) | 12.41 (10.01-15.13) | 20 (9-39) | 0.29 (0.13-0.59) | 994 (450-1888) | 11.2 (5.47-20.09) |
| Uruguay | 675 (539-820) | 15.31 (12.41-18.6) | 12 (10-18) | 0.25 (0.19-0.35) | 346 (268-456) | 9.23 (6.95-11.89) |
| USA | 78002 (65191-91731) | 18.12 (15.36-21.24) | 1022 (683-1191) | 0.27 (0.19-0.31) | 45531 (33049-52157) | 15.04 (11.05-17.13) |
| Uzbekistan | 4155 (3253-5139) | 14.88 (11.89-18) | 120 (84-212) | 0.54 (0.42-0.85) | 5282 (3784-8879) | 17.68 (13.1-28.46) |
| Vanuatu | 37 (29-45) | 15.31 (12.34-18.47) | 1 (0-1) | 0.36 (0.18-0.59) | 54 (27-86) | 17.87 (9.09-28.65) |
| Venezuela | 4037 (3235-4921) | 14.16 (11.46-17.13) | 26 (17-43) | 0.09 (0.06-0.15) | 999 (663-1659) | 3.57 (2.36-5.96) |
| Vietnam | 13329 (10518-16261) | 14.25 (11.44-17.23) | 298 (177-443) | 0.38 (0.21-0.59) | 9278 (5944-17407) | 10.77 (7.07-19.03) |
| Virgin Islands US | 20 (15-25) | 14.08 (11.39-17.03) | 1 (1-2) | 0.88 (0.55-1.2) | 31 (20-41) | 22.87 (15.54-31.09) |
| Yemen | 2731 (2141-3447) | 11.76 (9.49-14.33) | 90 (46-173) | 0.47 (0.23-1.09) | 5024 (2572-9155) | 17.03 (9-31.75) |
| Zambia | 1853 (1446-2294) | 14.59 (11.72-17.81) | 21 (9-43) | 0.16 (0.07-0.33) | 1373 (641-2636) | 7.72 (3.67-14.79) |
| Zimbabwe | 1606 (1265-1988) | 14.42 (11.6-17.6) | 65 (44-93) | 0.66 (0.45-0.95) | 3588 (2332-5283) | 25.73 (17.38-36.53) |
| ASIR, age-standardized incidence rate; ASMR, age-standardized mortality rate; DALYs, disability adjusted life year; ASDR, age-standardized DALY rate; UI, uncertainty interval. | | | | | | |
