# Supplementary material for: Global, Regional, and National Burden of Myocarditis in 204 Countries and Territories From 1990 to 2019: Updated Systematic Analysis
Source: JMIR Public Health Surveill. 2024 Jan 11;10:e46635. doi: 10.2196/46635 (PMC10811576; doi:10.2196/46635)
Supplement: Multimedia Appendix 2 [file publichealth_v10i1e46635_app2.docx]

| **Multimedia Appendix 2. Estimated annual percentage change in the burden rate of myocarditis in 204 countries and territories from 1990 to 2019.** | | | |
| --- | --- | --- | --- |
| **Location** | **EAPC of ASIR (95%CI)**  **from 1990 to 2019** | **EAPC of ASMR (95%CI)**  **from 1990 to 2019** | **EAPC of ASDR (95%CI)**  **from 1990 to 2019** |
| Afghanistan | -0.008 (-0.011 to -0.004) | 0.042 (-0.14 to 0.225) | -0.334 (-0.483 to -0.185) |
| Albania | 0.014 (0.003 to 0.026) | -2.199 (-2.571 to -1.827) | -2.075 (-2.363 to -1.785) |
| Algeria | 0.008 (0.005 to 0.011) | -0.975 (-1.071 to -0.879) | -1.453 (-1.528 to -1.377) |
| American Samoa | -0.017 (-0.018 to -0.015) | 0.293 (0.059 to 0.527) | 0.186 (0.002 to 0.37) |
| Andorra | -0.036 (-0.042 to -0.029) | -0.22 (-0.383 to -0.056) | -0.762 (-0.887 to -0.636) |
| Angola | -0.036 (-0.038 to -0.034) | -1.862 (-1.986 to -1.739) | -2.362 (-2.523 to -2.2) |
| Antigua and Barbuda | 0.032 (0.028 to 0.036) | 0.537 (0.446 to 0.628) | 0.295 (0.228 to 0.361) |
| Argentina | 0.005 (0.003 to 0.007) | -0.751 (-0.858 to -0.644) | -1.746 (-1.888 to -1.603) |
| Armenia | 0.007 (0.003 to 0.011) | 1.344 (0.73 to 1.962) | 0.467 (0.127 to 0.809) |
| Australia | 0.019 (0.018 to 0.021) | -1.011 (-1.568 to -0.451) | -1.335 (-1.809 to -0.858) |
| Austria | -0.061 (-0.083 to -0.039) | -0.633 (-0.898 to -0.366) | -1.297 (-1.467 to -1.127) |
| Azerbaijan | 0.025 (0.023 to 0.027) | -1.006 (-1.39 to -0.621) | -1.472 (-1.876 to -1.066) |
| Bahamas | 0.015 (0.013 to 0.016) | 0.84 (0.715 to 0.966) | 0.574 (0.479 to 0.669) |
| Bahrain | 0.04 (0.034 to 0.046) | -1.903 (-2.106 to -1.7) | -2.162 (-2.358 to -1.966) |
| Bangladesh | -0.034 (-0.039 to -0.028) | 0.305 (0.139 to 0.472) | 0.217 (0.084 to 0.35) |
| Barbados | 0.022 (0.02 to 0.024) | 0.462 (0.378 to 0.546) | 0.015 (-0.049 to 0.079) |
| Belarus | 0.01 (0.01 to 0.011) | -1.877 (-2.495 to -1.255) | -1.67 (-2.151 to -1.187) |
| Belgium | -0.009 (-0.061 to 0.043) | 0.314 (0.163 to 0.466) | -0.237 (-0.322 to -0.152) |
| Belize | -0.001 (-0.003 to 0.001) | 0.59 (0.325 to 0.855) | -0.025 (-0.217 to 0.167) |
| Benin | -0.007 (-0.009 to -0.004) | -1.431 (-1.587 to -1.275) | -0.801 (-0.909 to -0.693) |
| Bermuda | 0.016 (0.014 to 0.019) | 0.087 (0.001 to 0.174) | -0.068 (-0.195 to 0.06) |
| Bhutan | 0.026 (0.02 to 0.033) | 0.458 (0.335 to 0.582) | 0.167 (-0.019 to 0.353) |
| Bolivia | 0.017 (0.015 to 0.018) | -1.574 (-1.627 to -1.52) | -2.114 (-2.16 to -2.068) |
| Bosnia and Herzegovina | 0.011 (0.003 to 0.018) | -1.212 (-1.571 to -0.853) | -1.376 (-1.595 to -1.156) |
| Botswana | 0 (-0.005 to 0.005) | -1.269 (-1.479 to -1.059) | -0.615 (-0.75 to -0.48) |
| Brazil | -0.008 (-0.009 to -0.007) | 0.103 (-0.268 to 0.475) | -0.411 (-0.67 to -0.151) |
| Brunei | 0.001 (-0.004 to 0.007) | -0.917 (-1.478 to -0.352) | -1.381 (-1.915 to -0.843) |
| Bulgaria | 0.005 (0.002 to 0.007) | -0.313 (-1.135 to 0.517) | -0.766 (-1.484 to -0.042) |
| Burkina Faso | -0.007 (-0.009 to -0.005) | -0.954 (-1.135 to -0.771) | -0.307 (-0.52 to -0.094) |
| Burundi | 0.043 (0.04 to 0.045) | -2.221 (-2.429 to -2.012) | -2.427 (-2.631 to -2.222) |
| Cambodia | 0.016 (0.015 to 0.017) | -0.253 (-0.386 to -0.12) | -1.272 (-1.468 to -1.077) |
| Cameroon | -0.001 (-0.002 to -0.001) | -1.598 (-1.772 to -1.424) | -0.643 (-0.752 to -0.533) |
| Canada | 0.015 (0.013 to 0.017) | 2.014 (1.456 to 2.574) | 0.874 (0.479 to 1.271) |
| Cape Verde | 0.032 (0.028 to 0.037) | -0.518 (-0.734 to -0.301) | -0.814 (-0.942 to -0.685) |
| Central African Republic | -0.008 (-0.011 to -0.005) | -1.127 (-1.219 to -1.035) | -1.201 (-1.274 to -1.127) |
| Chad | 0.038 (0.037 to 0.039) | -1.325 (-1.419 to -1.23) | -0.375 (-0.422 to -0.328) |
| Chile | 0.016 (-0.022 to 0.053) | 0.775 (0.232 to 1.322) | 0.078 (-0.313 to 0.47) |
| China | -0.286 (-0.333 to -0.239) | -0.164 (-0.517 to 0.19) | -1.614 (-1.923 to -1.305) |
| Colombia | -0.019 (-0.02 to -0.018) | 0.659 (0.199 to 1.122) | 0.63 (0.162 to 1.099) |
| Comoros | -0.004 (-0.006 to -0.001) | -2.103 (-2.392 to -1.812) | -2.393 (-2.668 to -2.117) |
| Congo | 0.027 (0.025 to 0.03) | -2 (-2.208 to -1.792) | -2.199 (-2.405 to -1.992) |
| Cook Islands | -0.049 (-0.054 to -0.044) | -1.651 (-1.894 to -1.409) | -1.697 (-1.884 to -1.51) |
| Costa Rica | -0.023 (-0.024 to -0.021) | -0.645 (-0.921 to -0.367) | -0.98 (-1.262 to -0.697) |
| Croatia | -0.072 (-0.142 to -0.002) | -4.044 (-5.809 to -2.246) | -3.792 (-5.131 to -2.434) |
| Cuba | -0.01 (-0.011 to -0.008) | -0.924 (-1.086 to -0.762) | -1.562 (-1.751 to -1.372) |
| Cyprus | 0.032 (-0.01 to 0.074) | -1.464 (-1.579 to -1.349) | -1.959 (-2.126 to -1.792) |
| Czech | 0.005 (-0.006 to 0.016) | 1.126 (0.935 to 1.317) | -0.083 (-0.284 to 0.118) |
| Cote d'Ivoire | -0.004 (-0.007 to -0.001) | -1.44 (-1.588 to -1.291) | -0.808 (-0.933 to -0.682) |
| Denmark | 0.026 (-0.011 to 0.062) | 0.052 (-0.193 to 0.298) | -0.885 (-1.074 to -0.695) |
| Djibouti | 0.014 (0.013 to 0.016) | -1.868 (-1.981 to -1.755) | -2.054 (-2.2 to -1.908) |
| Dominica | 0.061 (0.057 to 0.065) | -0.221 (-0.322 to -0.121) | -0.291 (-0.383 to -0.198) |
| Dominican Republic | 0.005 (0.004 to 0.007) | 0.429 (0.005 to 0.854) | -0.752 (-1.082 to -0.421) |
| DR Congo | -0.018 (-0.023 to -0.012) | -1.125 (-1.183 to -1.066) | -1.699 (-1.787 to -1.611) |
| Ecuador | 0.037 (0.009 to 0.064) | -1.678 (-1.8 to -1.555) | -1.959 (-2.038 to -1.879) |
| Egypt | 0.037 (0.036 to 0.038) | -1.822 (-1.917 to -1.727) | -2.472 (-2.603 to -2.341) |
| El Salvador | -0.035 (-0.036 to -0.034) | 0.182 (-0.045 to 0.409) | -0.526 (-0.754 to -0.298) |
| Equatorial Guinea | 0.041 (0.033 to 0.049) | -3.325 (-3.686 to -2.964) | -3.664 (-3.961 to -3.366) |
| Eritrea | 0.014 (0.011 to 0.016) | -1.81 (-1.915 to -1.704) | -1.975 (-2.082 to -1.867) |
| Estonia | 0.027 (0.025 to 0.03) | 0.082 (-0.194 to 0.358) | -0.45 (-0.739 to -0.161) |
| Eswatini | -0.004 (-0.006 to -0.003) | -0.447 (-0.723 to -0.171) | -0.194 (-0.42 to 0.033) |
| Ethiopia | 0.007 (0 to 0.014) | -2.624 (-2.728 to -2.52) | -3.062 (-3.172 to -2.953) |
| Fiji | -0.013 (-0.016 to -0.011) | 0.608 (0.36 to 0.857) | 0.646 (0.434 to 0.859) |
| Finland | 0.032 (-0.019 to 0.083) | -1.064 (-1.328 to -0.799) | -1.464 (-1.73 to -1.198) |
| France | 0.02 (0.019 to 0.021) | 2.409 (1.863 to 2.959) | 1.139 (0.745 to 1.535) |
| Gabon | 0 (-0.001 to 0.001) | -1.773 (-1.862 to -1.684) | -1.977 (-2.065 to -1.888) |
| Gambia | -0.025 (-0.028 to -0.022) | -1.132 (-1.344 to -0.92) | -0.393 (-0.594 to -0.192) |
| Georgia | 0.011 (0.01 to 0.013) | 1.528 (0.77 to 2.292) | 1.228 (0.6 to 1.86) |
| Germany | 0.043 (0.037 to 0.048) | -1.147 (-1.387 to -0.906) | -0.95 (-1.155 to -0.744) |
| Ghana | -0.029 (-0.033 to -0.026) | -4.939 (-5.855 to -4.014) | -3.7 (-4.423 to -2.972) |
| Greece | -0.001 (-0.006 to 0.005) | -1.632 (-3.033 to -0.21) | -1.264 (-2.089 to -0.433) |
| Greenland | -0.002 (-0.007 to 0.003) | -0.649 (-1.07 to -0.226) | -1.389 (-1.783 to -0.994) |
| Grenada | 0.024 (0.014 to 0.034) | 0.282 (0.12 to 0.444) | -0.222 (-0.351 to -0.092) |
| Guam | -0.019 (-0.026 to -0.011) | -0.023 (-0.22 to 0.174) | 0.43 (0.191 to 0.669) |
| Guatemala | -0.047 (-0.051 to -0.044) | 0.435 (0.138 to 0.733) | 0.059 (-0.152 to 0.27) |
| Guinea | -0.004 (-0.008 to -0.001) | -1.235 (-1.368 to -1.102) | -0.488 (-0.602 to -0.374) |
| Guinea-Bissau | -0.016 (-0.017 to -0.014) | -1.394 (-1.524 to -1.263) | -0.827 (-0.898 to -0.756) |
| Guyana | -0.007 (-0.01 to -0.003) | 3.338 (2.749 to 3.93) | 3.089 (2.519 to 3.661) |
| Haiti | 0.003 (0 to 0.006) | -1.171 (-1.294 to -1.047) | -0.966 (-1.151 to -0.781) |
| Honduras | -0.011 (-0.016 to -0.005) | 0.539 (0.345 to 0.733) | -0.544 (-0.625 to -0.463) |
| Hungary | 0.009 (0.007 to 0.012) | 0.26 (-0.27 to 0.792) | -0.567 (-0.994 to -0.139) |
| Iceland | 0.025 (0.02 to 0.03) | -0.65 (-0.823 to -0.477) | -1.175 (-1.261 to -1.089) |
| India | -0.022 (-0.023 to -0.021) | -0.578 (-0.645 to -0.511) | -0.494 (-0.548 to -0.439) |
| Indonesia | 0.004 (0.004 to 0.004) | 0.601 (0.476 to 0.727) | -0.732 (-0.81 to -0.653) |
| Iran | -0.003 (-0.007 to 0.001) | -0.418 (-0.81 to -0.024) | -0.851 (-1.205 to -0.497) |
| Iraq | -0.004 (-0.006 to -0.002) | -0.151 (-0.253 to -0.049) | -0.989 (-1.117 to -0.861) |
| Ireland | 0.017 (0.015 to 0.02) | 4.906 (4.13 to 5.688) | 2.401 (1.923 to 2.881) |
| Israel | 0.013 (0.008 to 0.017) | -1.813 (-2.302 to -1.321) | -2.463 (-2.96 to -1.964) |
| Italy | -0.342 (-0.42 to -0.263) | 8.53 (5.199 to 11.966) | 3.561 (1.561 to 5.602) |
| Jamaica | 0.013 (0.011 to 0.015) | -0.164 (-0.543 to 0.216) | -0.637 (-0.97 to -0.302) |
| Japan | -0.254 (-0.303 to -0.204) | -1.161 (-1.389 to -0.933) | -1.094 (-1.408 to -0.78) |
| Jordan | 0.018 (0.015 to 0.021) | -2.683 (-2.917 to -2.447) | -2.575 (-2.842 to -2.308) |
| Kazakhstan | 0.003 (0.002 to 0.005) | 9.933 (7.292 to 12.639) | 8.702 (6.156 to 11.308) |
| Kenya | -0.018 (-0.022 to -0.014) | -0.556 (-0.686 to -0.425) | -1.033 (-1.18 to -0.885) |
| Kiribati | -0.012 (-0.014 to -0.01) | -0.537 (-0.58 to -0.495) | -0.505 (-0.544 to -0.466) |
| Kuwait | -0.03 (-0.047 to -0.012) | -0.459 (-0.745 to -0.172) | -1.032 (-1.287 to -0.777) |
| Kyrgyzstan | 0.009 (0.008 to 0.009) | -0.908 (-1.324 to -0.49) | -1.111 (-1.514 to -0.706) |
| Laos | 0.023 (0.02 to 0.026) | 0.615 (0.355 to 0.875) | 0.331 (0.023 to 0.64) |
| Latvia | -0.081 (-0.153 to -0.009) | -1.623 (-2.073 to -1.17) | -2.006 (-2.51 to -1.499) |
| Lebanon | -0.015 (-0.019 to -0.012) | -1.812 (-1.93 to -1.693) | -1.857 (-1.958 to -1.756) |
| Lesotho | -0.006 (-0.01 to -0.002) | 0.404 (0.227 to 0.581) | 0.563 (0.349 to 0.778) |
| Liberia | -0.001 (-0.003 to 0) | -1.737 (-1.987 to -1.487) | -1.143 (-1.43 to -0.856) |
| Libya | -0.012 (-0.016 to -0.009) | -1.862 (-1.954 to -1.771) | -2.39 (-2.471 to -2.31) |
| Lithuania | -0.045 (-0.099 to 0.01) | 1.118 (0.831 to 1.405) | 0.541 (0.234 to 0.848) |
| Luxembourg | -0.025 (-0.06 to 0.01) | -0.799 (-0.997 to -0.602) | -1.554 (-1.701 to -1.406) |
| Madagascar | -0.017 (-0.019 to -0.015) | -1.907 (-2.05 to -1.763) | -2.223 (-2.378 to -2.067) |
| Malawi | -0.027 (-0.03 to -0.025) | -1.901 (-2.116 to -1.686) | -2.546 (-2.779 to -2.312) |
| Malaysia | 0.027 (0.023 to 0.03) | -2.751 (-2.998 to -2.504) | -2.876 (-3.146 to -2.605) |
| Maldives | 0.032 (0.011 to 0.053) | -3.163 (-3.286 to -3.039) | -3.528 (-3.662 to -3.395) |
| Mali | 0.02 (0.017 to 0.023) | -1.687 (-1.901 to -1.472) | -0.922 (-1.125 to -0.719) |
| Malta | 0.034 (0.032 to 0.036) | -1.113 (-1.428 to -0.796) | -0.955 (-1.075 to -0.835) |
| Marshall Islands | 0.036 (0.032 to 0.04) | 0.33 (0.157 to 0.504) | 0.444 (0.249 to 0.639) |
| Mauritania | 0.019 (0.016 to 0.021) | -2.207 (-2.379 to -2.034) | -1.509 (-1.643 to -1.374) |
| Mauritius | 0.007 (0.005 to 0.009) | 0.847 (0.423 to 1.273) | 0.377 (0.089 to 0.666) |
| Mexico | -0.012 (-0.013 to -0.011) | 1.931 (1.764 to 2.099) | 1.386 (1.237 to 1.535) |
| Micronesia | -0.013 (-0.017 to -0.009) | -0.035 (-0.067 to -0.002) | -0.085 (-0.118 to -0.052) |
| Moldova | 0.019 (0.018 to 0.02) | -1.266 (-1.641 to -0.89) | -1.177 (-1.533 to -0.819) |
| Monaco | 0.02 (0.013 to 0.028) | -1.401 (-1.531 to -1.27) | -1.9 (-2.113 to -1.687) |
| Mongolia | -0.001 (-0.002 to 0.001) | -1.309 (-1.606 to -1.011) | -1.526 (-1.799 to -1.252) |
| Montenegro | 0.006 (0.002 to 0.01) | 0.832 (0.622 to 1.043) | -0.29 (-0.485 to -0.095) |
| Morocco | 0.014 (0.011 to 0.017) | -0.527 (-0.642 to -0.411) | -0.959 (-1.093 to -0.825) |
| Mozambique | -0.019 (-0.02 to -0.018) | -1.322 (-1.414 to -1.229) | -1.762 (-1.861 to -1.663) |
| Myanmar | -0.023 (-0.023 to -0.022) | -0.188 (-0.43 to 0.053) | -0.582 (-0.843 to -0.32) |
| Namibia | -0.015 (-0.016 to -0.015) | -1.249 (-1.379 to -1.119) | -0.904 (-0.971 to -0.837) |
| Nauru | -0.028 (-0.041 to -0.015) | 0.308 (-0.116 to 0.735) | 0.386 (-0.058 to 0.832) |
| Nepal | -0.03 (-0.034 to -0.025) | 0.606 (0.523 to 0.689) | 0.218 (0.161 to 0.275) |
| Netherlands | 0.034 (0.031 to 0.036) | -2.81 (-3.252 to -2.366) | -2.84 (-3.15 to -2.53) |
| New Zealand | -0.226 (-0.33 to -0.121) | -0.429 (-0.716 to -0.142) | -1.029 (-1.284 to -0.774) |
| Nicaragua | -0.001 (-0.005 to 0.003) | -0.654 (-0.886 to -0.421) | -1.41 (-1.542 to -1.277) |
| Niger | -0.013 (-0.017 to -0.008) | -1.649 (-1.835 to -1.463) | -1.178 (-1.356 to -1.001) |
| Nigeria | -0.028 (-0.038 to -0.017) | -2.844 (-3.126 to -2.561) | -1.951 (-2.182 to -1.719) |
| Niue | 0.007 (0.002 to 0.013) | -0.55 (-0.65 to -0.451) | -0.458 (-0.606 to -0.31) |
| North Korea | 0.06 (0.057 to 0.063) | 0.6 (0.288 to 0.912) | -0.124 (-0.331 to 0.083) |
| North Macedonia | 0.01 (0.005 to 0.014) | -0.332 (-0.732 to 0.07) | -1.449 (-1.986 to -0.91) |
| Northern Mariana Islands | -0.023 (-0.038 to -0.008) | 1.01 (0.534 to 1.489) | 0.927 (0.5 to 1.355) |
| Norway | -0.011 (-0.029 to 0.006) | 0.084 (-0.259 to 0.428) | -0.78 (-1.07 to -0.489) |
| Oman | 0.05 (0.032 to 0.067) | -0.655 (-0.893 to -0.415) | -0.552 (-0.861 to -0.243) |
| Pakistan | -0.028 (-0.029 to -0.027) | 0.058 (-0.074 to 0.191) | 0.395 (0.285 to 0.505) |
| Palau | 0.029 (0.014 to 0.045) | -0.532 (-0.612 to -0.452) | -0.49 (-0.571 to -0.408) |
| Palestine | 0.008 (0.006 to 0.01) | 1.188 (0.702 to 1.675) | 0.202 (-0.122 to 0.528) |
| Panama | -0.012 (-0.013 to -0.01) | -0.409 (-0.621 to -0.197) | -0.289 (-0.45 to -0.127) |
| Papua New Guinea | 0.008 (0.006 to 0.01) | 0.647 (0.536 to 0.759) | 0.611 (0.486 to 0.736) |
| Paraguay | 0.004 (0.003 to 0.004) | 1.688 (1.398 to 1.979) | 1.008 (0.717 to 1.3) |
| Peru | 0 (-0.001 to 0.002) | -4.184 (-4.555 to -3.811) | -4.102 (-4.464 to -3.74) |
| Philippines | -0.089 (-0.104 to -0.073) | 1.078 (0.738 to 1.419) | 2.327 (1.801 to 2.855) |
| Poland | 0.003 (-0.058 to 0.064) | -1.011 (-1.325 to -0.695) | -1.783 (-1.971 to -1.595) |
| Portugal | 0.061 (0.053 to 0.068) | 0.082 (-0.268 to 0.434) | -1.566 (-1.864 to -1.267) |
| Puerto Rico | -0.004 (-0.006 to -0.002) | -1.134 (-1.676 to -0.589) | -1.588 (-2.121 to -1.053) |
| Qatar | 0.134 (0.12 to 0.149) | -2.789 (-3.073 to -2.504) | -3.42 (-3.601 to -3.239) |
| Romania | -0.127 (-0.173 to -0.081) | -0.063 (-0.378 to 0.253) | 0.735 (0.434 to 1.038) |
| Russia | 0.016 (0.015 to 0.017) | 0.385 (0.178 to 0.592) | 0.718 (0.5 to 0.935) |
| Rwanda | -0.016 (-0.023 to -0.008) | -2.668 (-2.879 to -2.457) | -2.87 (-3.064 to -2.676) |
| Saint Kitts and Nevis | 0.031 (0.029 to 0.034) | 0.232 (0.117 to 0.348) | -0.058 (-0.188 to 0.073) |
| Saint Lucia | 0.034 (0.033 to 0.036) | -0.013 (-0.22 to 0.195) | 0.201 (0.083 to 0.32) |
| Saint Vincent and the Grenadines | 0.051 (0.049 to 0.053) | 0.721 (0.62 to 0.822) | 0.132 (-0.037 to 0.301) |
| Samoa | -0.003 (-0.003 to -0.002) | -0.149 (-0.248 to -0.049) | -0.224 (-0.303 to -0.144) |
| San Marino | -0.014 (-0.018 to -0.01) | 0.754 (0.617 to 0.891) | 0.21 (0.091 to 0.33) |
| Sao Tome and Principe | 0.018 (0.017 to 0.018) | -1.115 (-1.335 to -0.894) | -0.414 (-0.609 to -0.219) |
| Saudi Arabia | 0.013 (0.005 to 0.022) | -3.551 (-3.914 to -3.187) | -3.774 (-4.145 to -3.4) |
| Senegal | -0.004 (-0.004 to -0.003) | -0.983 (-1.123 to -0.843) | -0.267 (-0.463 to -0.071) |
| Serbia | -0.091 (-0.127 to -0.056) | -3.226 (-3.448 to -3.005) | -4.696 (-4.997 to -4.394) |
| Seychelles | 0.051 (0.044 to 0.058) | -1.243 (-1.409 to -1.076) | -1.073 (-1.27 to -0.876) |
| Sierra Leone | 0.003 (-0.002 to 0.007) | -1.015 (-1.168 to -0.861) | -0.202 (-0.357 to -0.047) |
| Singapore | 0.024 (0.016 to 0.033) | -3.659 (-4.332 to -2.982) | -4.034 (-4.696 to -3.367) |
| Slovakia | -0.136 (-0.182 to -0.09) | -1.74 (-1.978 to -1.501) | -2.32 (-2.572 to -2.066) |
| Slovenia | -0.006 (-0.035 to 0.023) | 3.398 (2.783 to 4.017) | 1.273 (0.714 to 1.835) |
| Solomon Islands | -0.037 (-0.039 to -0.036) | 0.297 (0.21 to 0.384) | 0.155 (0.068 to 0.243) |
| Somalia | -0.029 (-0.032 to -0.025) | -1.413 (-1.621 to -1.205) | -1.505 (-1.747 to -1.263) |
| South Africa | 0.003 (-0.002 to 0.008) | -3.163 (-3.577 to -2.747) | -3.549 (-3.878 to -3.218) |
| South Korea | 0.059 (0.055 to 0.063) | -4.238 (-5.196 to -3.27) | -3.478 (-4.022 to -2.931) |
| South Sudan | -0.027 (-0.032 to -0.021) | -2.328 (-2.66 to -1.995) | -2.477 (-2.845 to -2.107) |
| Spain | 0.029 (0.025 to 0.034) | 1.83 (1.444 to 2.218) | 0.576 (0.296 to 0.858) |
| Sri Lanka | -0.047 (-0.051 to -0.042) | -0.95 (-1.122 to -0.777) | -1.436 (-1.75 to -1.121) |
| Sudan | 0.017 (0.017 to 0.018) | -0.739 (-0.817 to -0.66) | -1.112 (-1.217 to -1.008) |
| Suriname | -0.016 (-0.017 to -0.014) | -0.535 (-0.678 to -0.391) | -0.839 (-0.977 to -0.702) |
| Sweden | -0.019 (-0.052 to 0.014) | 1.663 (0.668 to 2.669) | 1.005 (0.131 to 1.886) |
| Switzerland | -0.052 (-0.11 to 0.006) | -0.469 (-0.723 to -0.214) | -1.533 (-1.768 to -1.299) |
| Syria | -0.022 (-0.036 to -0.008) | -1.468 (-1.669 to -1.266) | -2.028 (-2.349 to -1.706) |
| Taiwan toChina | -0.152 (-0.189 to -0.115) | 4.05 (3.543 to 4.559) | 3.021 (2.591 to 3.452) |
| Tajikistan | 0.012 (0.01 to 0.013) | 0.069 (-0.122 to 0.26) | -0.454 (-0.637 to -0.27) |
| Tanzania | -0.004 (-0.005 to -0.003) | -1.953 (-2.099 to -1.806) | -1.937 (-2.066 to -1.807) |
| Thailand | 0.006 (0.004 to 0.007) | -4.636 (-5.152 to -4.117) | -4.242 (-4.84 to -3.64) |
| Timor-Leste | -0.016 (-0.02 to -0.013) | 1.109 (1.004 to 1.215) | 0.549 (0.442 to 0.657) |
| Togo | -0.03 (-0.031 to -0.028) | -1.306 (-1.499 to -1.113) | -0.636 (-0.769 to -0.503) |
| Tokelau | 0.045 (0.036 to 0.053) | -0.627 (-0.724 to -0.53) | -0.639 (-0.774 to -0.504) |
| Tonga | -0.016 (-0.02 to -0.013) | -0.043 (-0.137 to 0.051) | 0.032 (-0.093 to 0.158) |
| Trinidad and Tobago | 0.008 (0.006 to 0.009) | -1.941 (-2.447 to -1.432) | -1.558 (-1.978 to -1.135) |
| Tunisia | -0.017 (-0.018 to -0.016) | -1.207 (-1.28 to -1.135) | -1.785 (-1.853 to -1.717) |
| Turkey | 0.031 (0.022 to 0.04) | -2.354 (-2.511 to -2.198) | -2.689 (-2.789 to -2.59) |
| Turkmenistan | 0.028 (0.027 to 0.03) | 0.688 (0.15 to 1.228) | 0.706 (0.229 to 1.185) |
| Tuvalu | 0.075 (0.072 to 0.079) | -0.658 (-0.721 to -0.594) | -0.901 (-0.967 to -0.836) |
| Uganda | -0.027 (-0.031 to -0.023) | -1.416 (-1.56 to -1.271) | -1.494 (-1.648 to -1.34) |
| UK | -0.126 (-0.215 to -0.037) | 2.702 (2.024 to 3.383) | 1.707 (1.148 to 2.268) |
| Ukraine | 0.014 (0.013 to 0.014) | 1.791 (1.276 to 2.309) | 1.906 (1.328 to 2.487) |
| United Arab Emirates | 0.067 (0.05 to 0.083) | -0.949 (-1.262 to -0.635) | -1.111 (-1.243 to -0.978) |
| Uruguay | -0.007 (-0.007 to -0.006) | -0.373 (-0.58 to -0.166) | -1.044 (-1.211 to -0.876) |
| USA | -0.927 (-1.127 to -0.727) | 1.747 (1.162 to 2.336) | 1.312 (0.8 to 1.826) |
| Uzbekistan | 0.006 (0.004 to 0.009) | 3.452 (3.16 to 3.745) | 2.884 (2.586 to 3.182) |
| Vanuatu | -0.025 (-0.027 to -0.023) | 0.753 (0.639 to 0.867) | 0.828 (0.695 to 0.962) |
| Venezuela | -0.001 (-0.004 to 0.001) | -3.547 (-4.328 to -2.76) | -3.154 (-3.908 to -2.393) |
| Vietnam | 0.024 (0.022 to 0.026) | -0.292 (-0.461 to -0.122) | -0.712 (-0.876 to -0.548) |
| Virgin Islands US | -0.005 (-0.007 to -0.003) | 0.632 (0.298 to 0.967) | -0.194 (-0.632 to 0.246) |
| Yemen | 0.02 (0.016 to 0.025) | -0.2 (-0.285 to -0.116) | -0.416 (-0.506 to -0.327) |
| Zambia | -0.029 (-0.031 to -0.026) | -1.321 (-1.465 to -1.176) | -2.092 (-2.223 to -1.962) |
| Zimbabwe | -0.031 (-0.035 to -0.027) | 1.164 (0.915 to 1.413) | 1.554 (1.27 to 1.84) |
| EAPC, estimated annual percentage change; ASIR, age-standardized incidence rate; ASMR, age-standardized mortality rate; DALYs, disability adjusted life year; ASDR, age-standardized DALY rate; CI, confidential interval. | | | |
