# Supplementary figures and images for: Global, Regional, and National Burden of Myocarditis in 204 Countries and Territories From 1990 to 2019: Updated Systematic Analysis
Source: JMIR Public Health Surveill. 2024 Jan 11;10:e46635. doi: 10.2196/46635 (PMC10811576; doi:10.2196/46635)

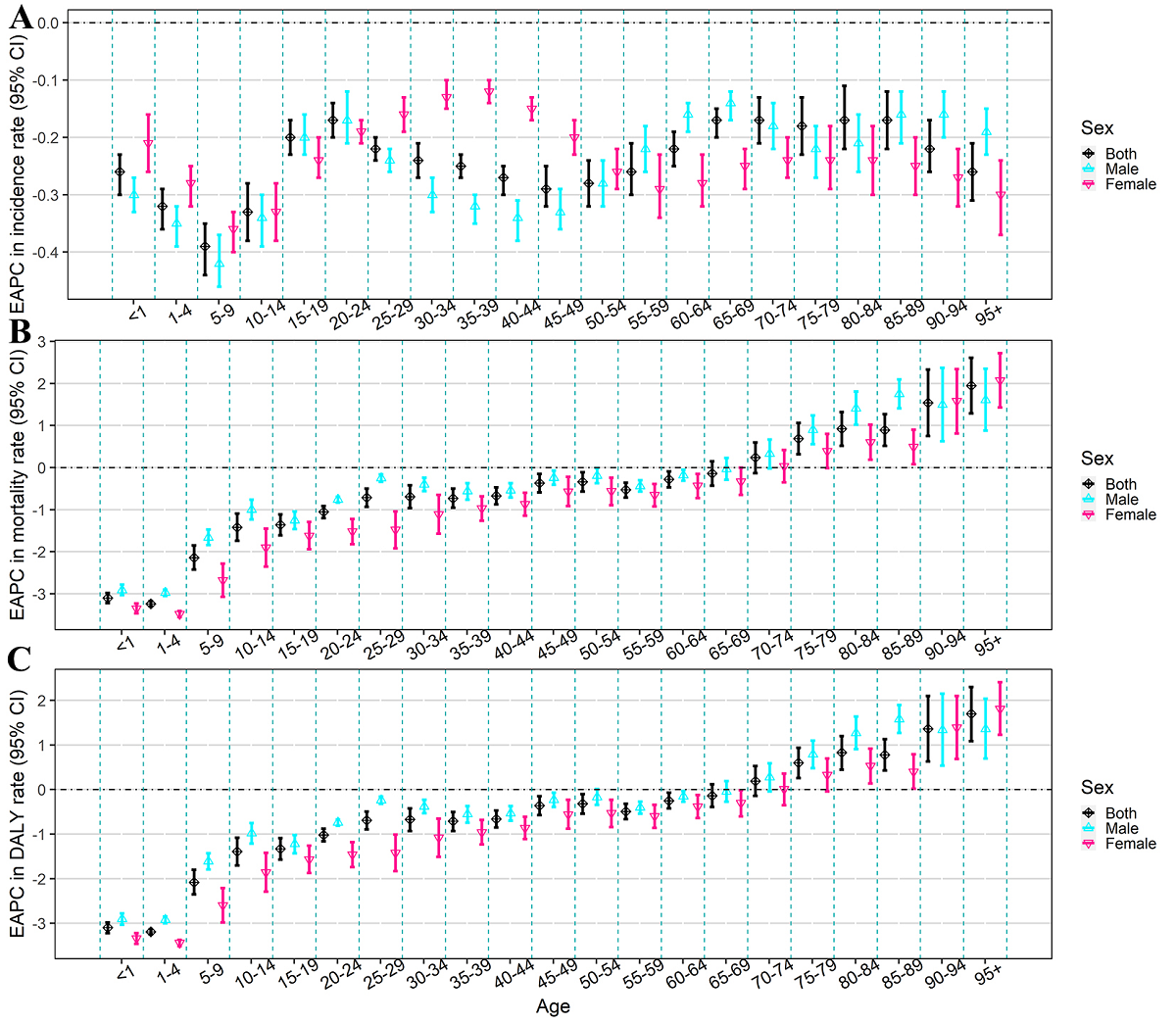

Supplement: Multimedia Appendix 3 [file publichealth_v10i1e46635_app3.png]

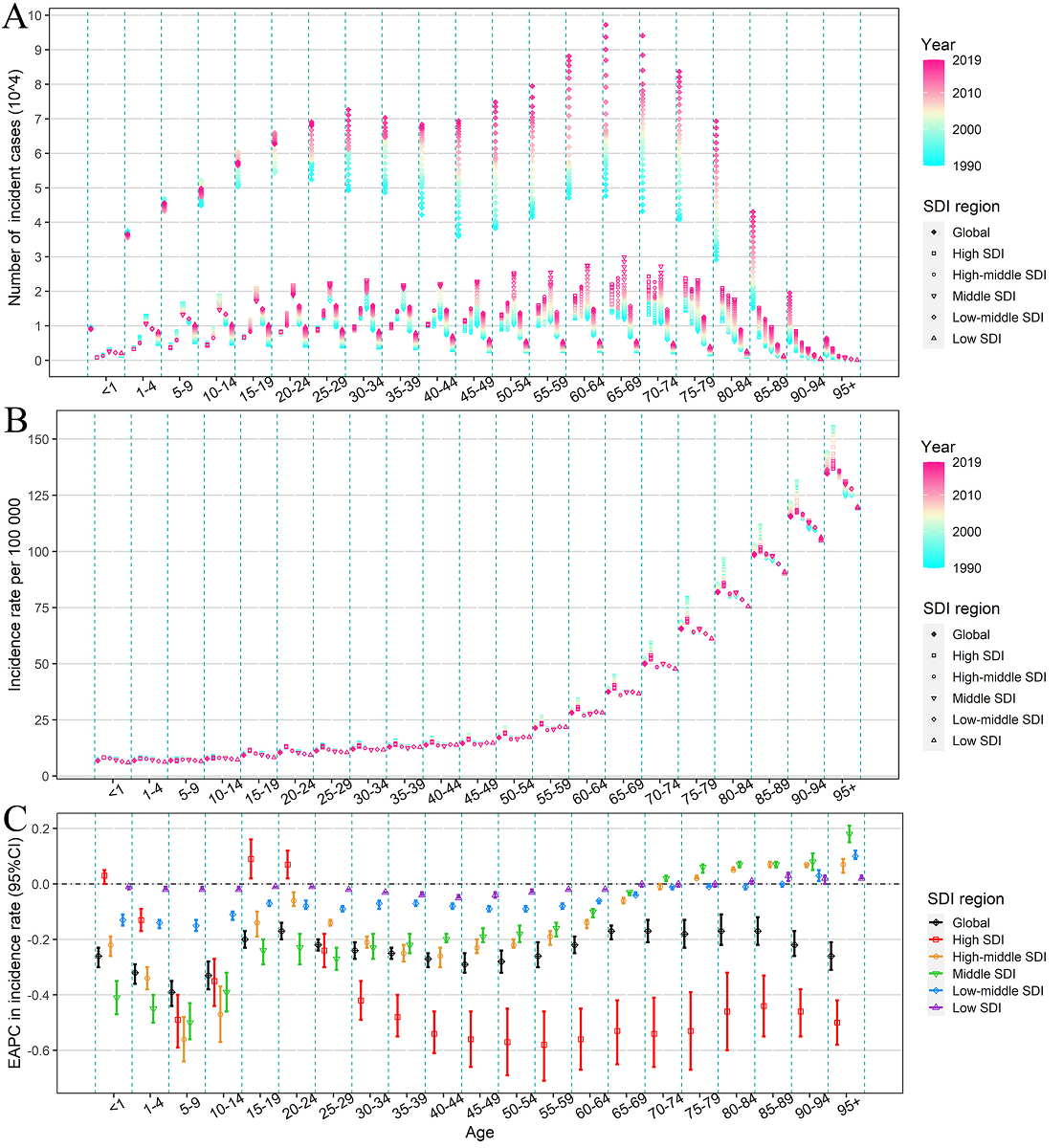

Supplement: Multimedia Appendix 4 [file publichealth_v10i1e46635_app4.png]

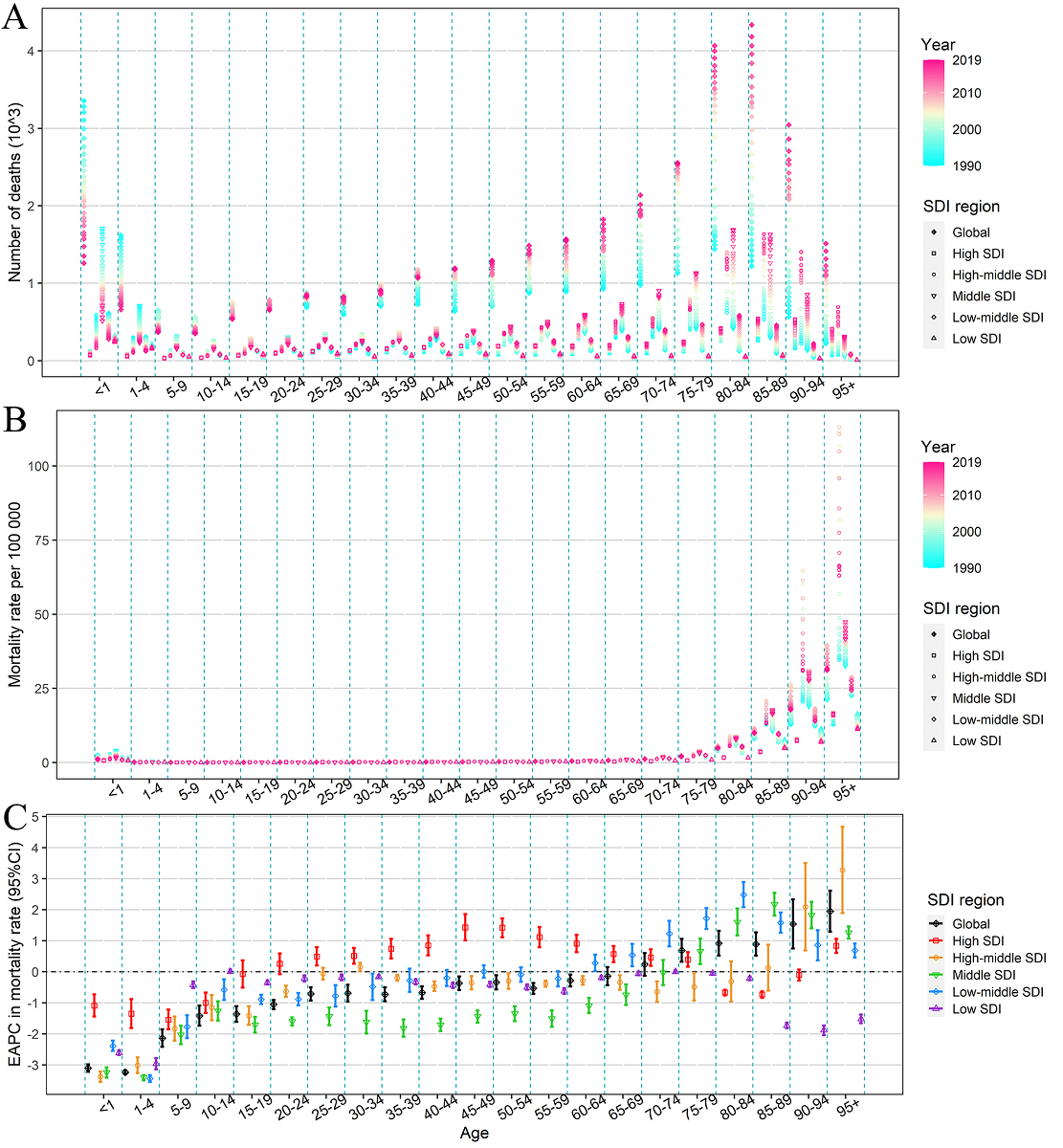

Supplement: Multimedia Appendix 5 [file publichealth_v10i1e46635_app5.png]

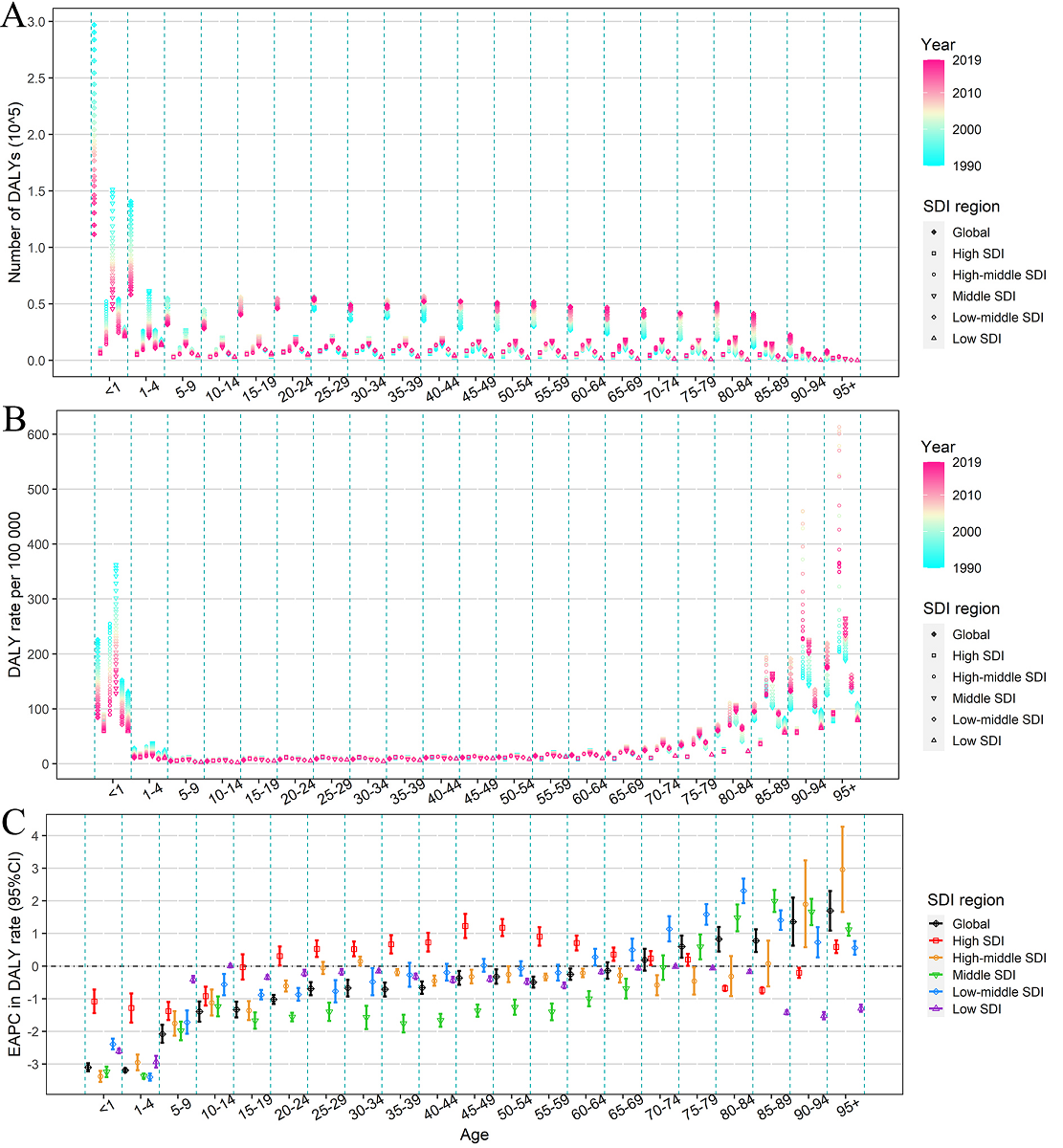

Supplement: Multimedia Appendix 6 [file publichealth_v10i1e46635_app6.png]

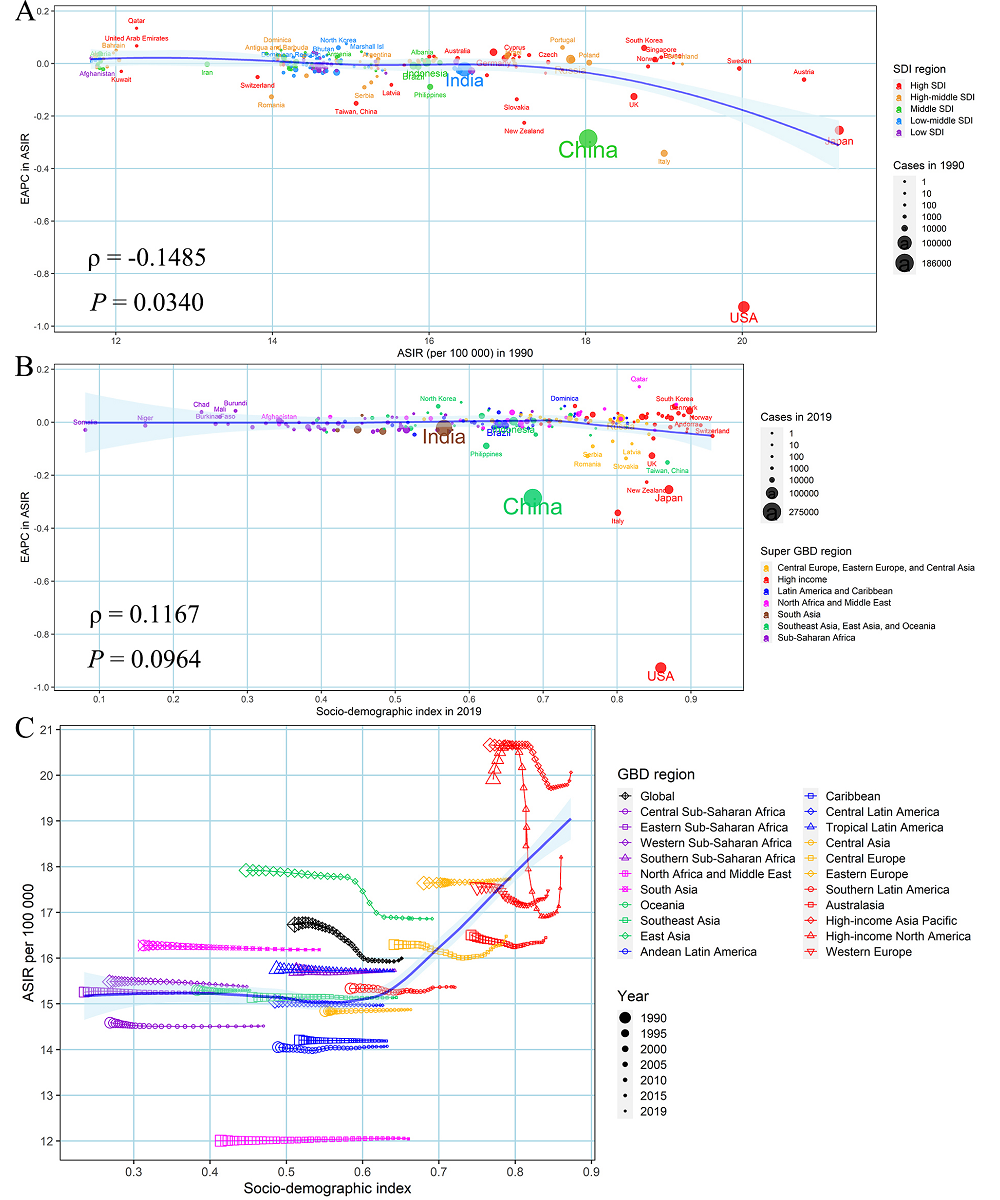

Supplement: Multimedia Appendix 7 [file publichealth_v10i1e46635_app7.png]

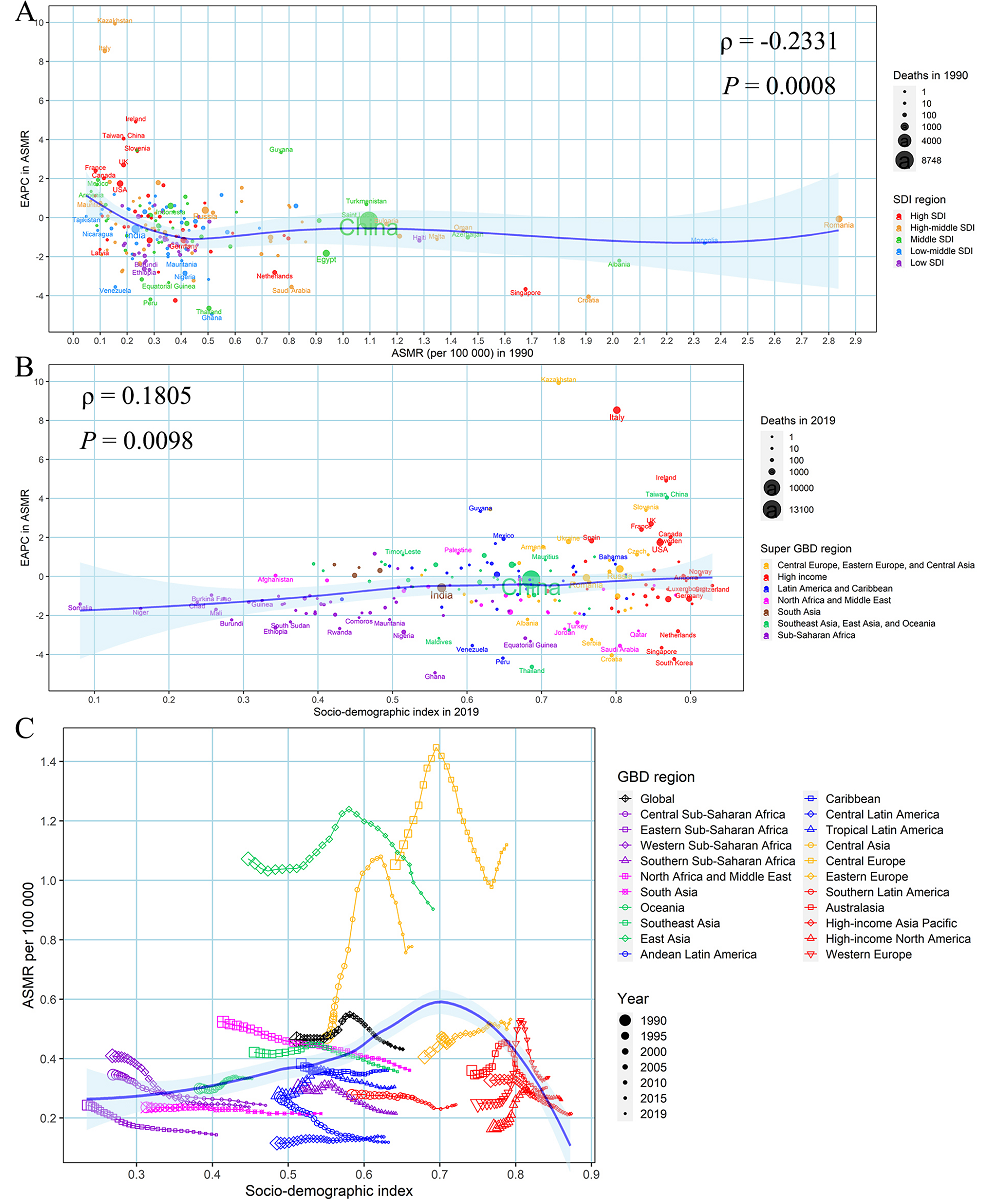

Supplement: Multimedia Appendix 8 [file publichealth_v10i1e46635_app8.png]

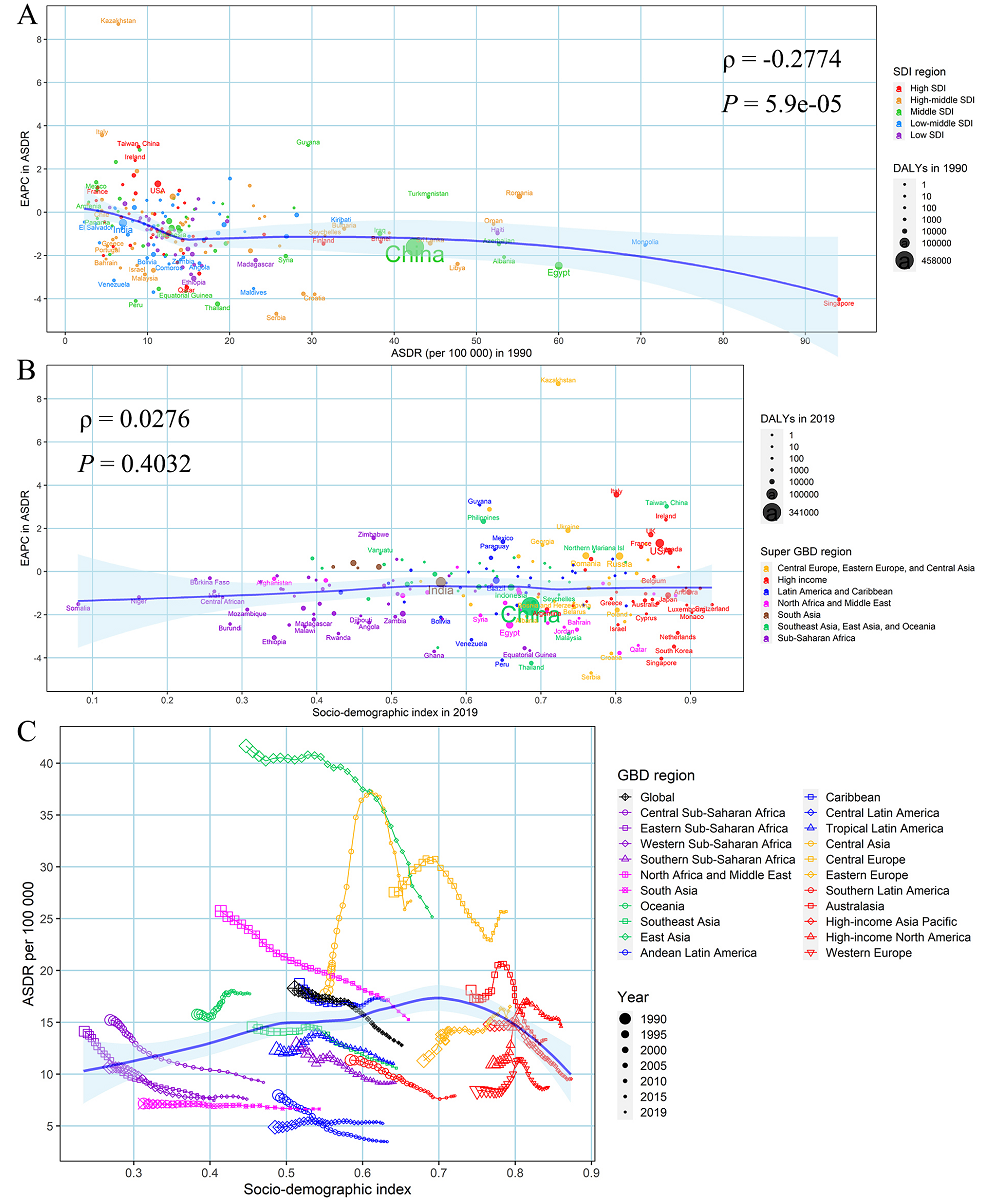

Supplement: Multimedia Appendix 9 [file publichealth_v10i1e46635_app9.png]
